# Post-discharge malaria chemoprevention in children admitted with severe anaemia in malaria-endemic settings in Africa: a systematic review and individual patient data meta-analysis of randomised controlled trials

**DOI:** 10.1016/S2214-109X(23)00492-8

**Published:** 2023-12-12

**Authors:** Kamija S Phiri, Carole Khairallah, Titus K Kwambai, Kalifa Bojang, Aggrey Dhabangi, Robert Opoka, Richard Idro, Kasia Stepniewska, Michael Boele van Hensbroek, Chandy C John, Bjarne Robberstad, Brian Greenwood, Feiko O ter Kuile

**Affiliations:** a School of Global and Public Health, Kamuzu University of Health Sciences (KUHeS), Blantyre, Malawi; b Training and Research Unit of Excellence, Blantyre, Malawi; c Department of Clinical Sciences, Liverpool School of Tropical Medicine, Liverpool, UK; d Division of Parasitic Diseases and Malaria, Global Health Center, Centers for Disease Control and Prevention, Kisumu, Kenya; e Centre for Global Health Research, Kenya Medical Research Institute, Kisumu, Kenya; f Medical Research Council Unit The Gambia at the London School of Hygiene and Tropical Medicine, Fajara, The Gambia; g Makerere University College of Health Sciences, Kampala, Uganda; h Aga Khan University, Medical College, Nairobi, Kenya; i Worldwide Antimalarial Resistance Network (WWARN), Oxford, UK; j Centre for Tropical Medicine and Global Health, Nuffield Department of Clinical Medicine, University of Oxford, Oxford, UK; k Infectious Diseases Data Observatory (IDDO), Oxford, UK; l Amsterdam Centre for Global Child Health, Emma Children's Hospital, Amsterdam UMC, University of Amsterdam, Amsterdam, Netherlands; m Ryan White Center for Pediatric Infectious Disease and Global Health, Indiana University School of Medicine, Indianapolis, IN, USA; n Section for Ethics and Health Economics, Department of Global Public Health and Primary Care, University of Bergen, Bergen, Norway; o Faculty of Infectious and Tropical Diseases, London School of Hygiene & Tropical Medicine, London, UK

## Abstract

**Background:**

Severe anaemia is associated with high in-hospital mortality among young children. In malaria-endemic areas, surviving children also have an increased risk of mortality or readmission after hospital discharge. We conducted a systematic review and individual patient data meta-analysis to determine the efficacy of monthly post-discharge malaria chemoprevention in children recovering from severe anaemia.

**Methods:**

This analysis was conducted according to PRISMA-IPD guidelines. We searched multiple databases on Aug 28, 2023, without date or language restrictions, for randomised controlled trials comparing monthly post-discharge malaria chemoprevention with placebo or standard of care among children (aged <15 years) admitted with severe anaemia in malaria-endemic Africa. Trials using daily or weekly malaria prophylaxis were not eligible. The investigators from all eligible trials shared pseudonymised datasets, which were standardised and merged for analysis. The primary outcome was all-cause mortality during the intervention period. Analyses were performed in the modified intention-to-treat population, including all randomly assigned participants who contributed to the endpoint. Fixed-effects two-stage meta-analysis of risk ratios (RRs) was used to generate pooled effect estimates for mortality. Recurrent time-to-event data (readmissions or clinic visits) were analysed using one-stage mixed-effects Prentice-Williams-Peterson total-time models to obtain hazard ratios (HRs). This study is registered with PROSPERO, CRD42022308791.

**Findings:**

Our search identified 91 articles, of which 78 were excluded by title and abstract, and a further ten did not meet eligibility criteria. Three double-blind, placebo-controlled trials, including 3663 children with severe anaemia, were included in the systematic review and meta-analysis; 3507 (95·7%) contributed to the modified intention-to-treat analysis. Participants received monthly sulfadoxine–pyrimethamine until the end of the malaria transmission season (mean 3·1 courses per child [range 1–6]; n=1085; The Gambia), monthly artemether–lumefantrine given at the end of weeks 4 and 8 post discharge (n=1373; Malawi), or monthly dihydroartemisinin–piperaquine given at the end of weeks 2, 6, and 10 post discharge (n=1049; Uganda and Kenya). During the intervention period, post-discharge malaria chemoprevention was associated with a 77% reduction in mortality (RR 0·23 [95% CI 0·08–0·70], p=0·0094, *I*^2^=0%) and a 55% reduction in all-cause readmissions (HR 0·45 [95% CI 0·36–0·56], p<0·0001) compared with placebo. The protective effect was restricted to the intervention period and was not sustained after the direct pharmacodynamic effect of the drugs had waned. The small number of trials limited our ability to assess heterogeneity, its sources, and publication bias.

**Interpretation:**

In malaria-endemic Africa, post-discharge malaria chemoprevention reduces mortality and readmissions in recently discharged children recovering from severe anaemia. Post-discharge malaria chemoprevention could be a valuable strategy for the management of this group at high risk. Future research should focus on methods of delivery, options to prolong the protection duration, other hospitalised groups at high risk, and interventions targeting non-malarial causes of post-discharge morbidity.

**Funding:**

The Research-Council of Norway and the Bill-&-Melinda-Gates-Foundation through the Worldwide-Antimalarial-Research-Network.

## Introduction

In sub-Saharan Africa, severe anaemia is associated with high in-hospital mortality among children younger than 5 years.^1^, ^2^, ^3^, ^4^ However, in malaria-endemic areas, surviving children with severe anaemia also remain at an increased risk of mortality or readmission for at least 6 months after hospital discharge.^5^, ^6^ In June 2022, WHO recommended post-discharge malaria chemoprevention for children recently discharged from hospital after recovery from severe anaemia.^7^ The recommendation was based on the results of several promising trials in highly malaria-endemic areas of Africa, showing that monthly treatment courses of sulfadoxine–pyrimethamine^8^ or artemisinin-based combination therapies^9^, ^10^ prevented a substantial number of post-discharge deaths and readmissions. Here, we present the systematic review and meta-analysis that was a core part of the evidence that led to this WHO recommendation. The pooled evidence could support policy makers in introducing post-discharge malaria chemoprevention for the management of severe anaemia in malaria-endemic areas in Africa.

**Research in context**

**Evidence before this study**

Several trials have shown that post-discharge malaria chemoprevention with monthly treatment doses of antimalarials can reduce the risk of death, hospital readmissions, and outpatient clinic visits. We searched PubMed, Scopus, Embase, Web of Science, Cochrane Central Register of Controlled Trials, and the WHO International Clinical Trials Registry Platform from database inception to Jan 31, 2022, without language restrictions, for randomised controlled trials assessing the impact of post-discharge malaria chemoprevention for the post-discharge management of children with severe anaemia in malaria-endemic areas. The following search terms were used in PubMed: (child OR childhood OR infant OR pediatric OR paediatric) AND (malaria OR plasmodium) AND (“severe anaemia” OR “severe anemia” OR transfusion) AND (recurrence OR discharge OR postdischarge OR post-discharge). The search identified only three such trials, all of which were placebo controlled. An updated literature search on Aug 28, 2023, identified no additional studies. No previous meta-analysis was identified that addressed the impact of post-discharge malaria chemoprevention in hospitalised children with severe anaemia.

**Added value of this study**

This is the first meta-analysis of post-discharge malaria chemoprevention. The analysis included data from 3507 children with severe anaemia and confirmed that post-discharge malaria chemoprevention effectively reduces death and readmissions post discharge. The benefits were evident regardless of bednet use and greatest among those admitted with malaria-associated anaemia, but also evident among those admitted with other causes of severe anaemia.

**Implications of all the available evidence**

The available evidence, together with cost-effectiveness, delivery mechanism, and modelling studies, support the WHO malaria chemoprevention guidelines updated in June, 2022, which now recommend post-discharge malaria chemoprevention for the care of hospitalised children with severe anaemia living in settings with moderate to high malaria transmission.

## Methods

### Search strategy and selection criteria

We conducted a systematic review and individual patient data (IPD) meta-analysis of randomised controlled trials evaluating monthly post-discharge malaria chemoprevention in children recovering from severe anaemia. The analysis followed the PRISMA-IPD statement.^11^ We identified eligible studies by performing a literature search in PubMed, Scopus, Embase, Web of Science, Cochrane Central Register of Controlled Trials, and the WHO International Clinical Trials Registry Platform on Jan 31, 2022, and again on Aug 28, 2023, without date or language restrictions (appendix p 3). The following search terms were used in PubMed: (child OR childhood OR infant OR pediatric OR paediatric) AND (malaria OR plasmodium) AND (“severe anaemia” OR “severe anemia” OR transfusion) AND (recurrence OR discharge OR postdischarge OR post-discharge). In addition, we identified other relevant studies by scanning reference lists of all identified articles and searching in Google and Google Scholar. Randomised controlled trials were eligible if they compared monthly malaria chemoprevention regimens after discharge against a placebo or the current standard of post-discharge care in a malaria-endemic area of Africa among children younger than 15 years recently discharged after hospitalisation for severe anaemia. Trials using daily or weekly malaria prophylaxis were not eligible.

Two independent reviewers (TKK and FOtK) screened titles, abstracts, and full texts of all identified citations and agreed on the final eligibility. Disagreements between reviewers were resolved by CK. Reviewers were unmasked to the authors of the source study. Two reviewers (TKK and FOtK) independently assessed the risk of bias for the included trials using the Cochrane risk-of-bias tool for randomised trials, version 2 (appendix p 3).^12^ The investigators from all eligible trials shared pseudonymised datasets, which were standardised and merged for analysis. The study protocol is available online. The original studies were approved by the relevant local and international partner ethics committees and institutional review boards.

### Data analysis

The primary outcome was all-cause mortality during the intervention period. Secondary outcomes were all-cause and cause-specific readmissions; non-severe, all-cause sick-child clinical visits; episodes of uncomplicated clinical malaria (any, or those associated with parasite densities ≥5000 parasites per μL); and clinic visits for any illness unrelated to malaria (appendix p 3).

Analyses were performed in the modified intention-to-treat population, including all randomly assigned participants who contributed to the endpoint. Recurrent time-to-event data (readmissions and clinic visits) were analysed using one-stage mixed-effects Prentice-Williams-Peterson total-time models to obtain hazard ratios (HRs; appendix p 4).^13^ Each IPD model included study site (multiple sites per study) as a random effect and the bodyweight category used at randomisation as a fixed-effect covariate to adjust for stratification factors. The adjusted models included five additional covariables available for all studies, including previous hospitalisation (yes or no), bednet use (yes or no), cubic of age in months, dose in mg/kg (tercile categories), and sex (male or female), because in previous studies these were found to be predictive of the rate of readmissions.^9^, ^10^ For one of the studies, only aggregated mortality data were available without time-to-death information.^8^ The impact on mortality data was therefore analysed using fixed-effects two-stage meta-analyses of risk ratios (RRs; appendix p 3). Random-effects models for this mortality analysis were not considered because the between-study variance cannot be reliably estimated with few studies.^14^ The analysis was stratified a priori by the post-discharge malaria chemoprevention intervention period (primary analysis) and post-intervention period to assess the direct effect of the intervention and any rebound or delayed episodes after the direct pharmacological protective effect of the antimalarial drugs had waned. Because the proportional-hazards assumptions were violated for most endpoints when assessing the cumulative effect over the entire follow-up period, the incidence rate ratio (IRR), the absolute risk difference, and its inverse, the number needed to treat (NNT), were calculated post hoc using negative binomial regression (appendix p 6). p<0·05 was considered to indicate statistical significance (two-sided tests). Effect modification was assessed on the additive scale as the relative excess risk due to interaction (RERI) and on the multiplicative scale as the ratio of ratios (appendix p 6). Heterogeneity for mortality was measured using the *I*^2^ statistic (appendix p 6).^6^ Further sensitivity analyses to assess robustness were conducted using alternative time-to-event models and count models (appendix p 4). Data were analysed using STATA/MP version 17.0 and RStudio version 4.2.1 (2022-06-23).

This study is registered with PROSPERO, CRD42022308791.

### Role of the funding source

The funders of the study had no role in study design, data collection, data analysis, data interpretation, or writing of the report.

## Results

**Figure 1:**
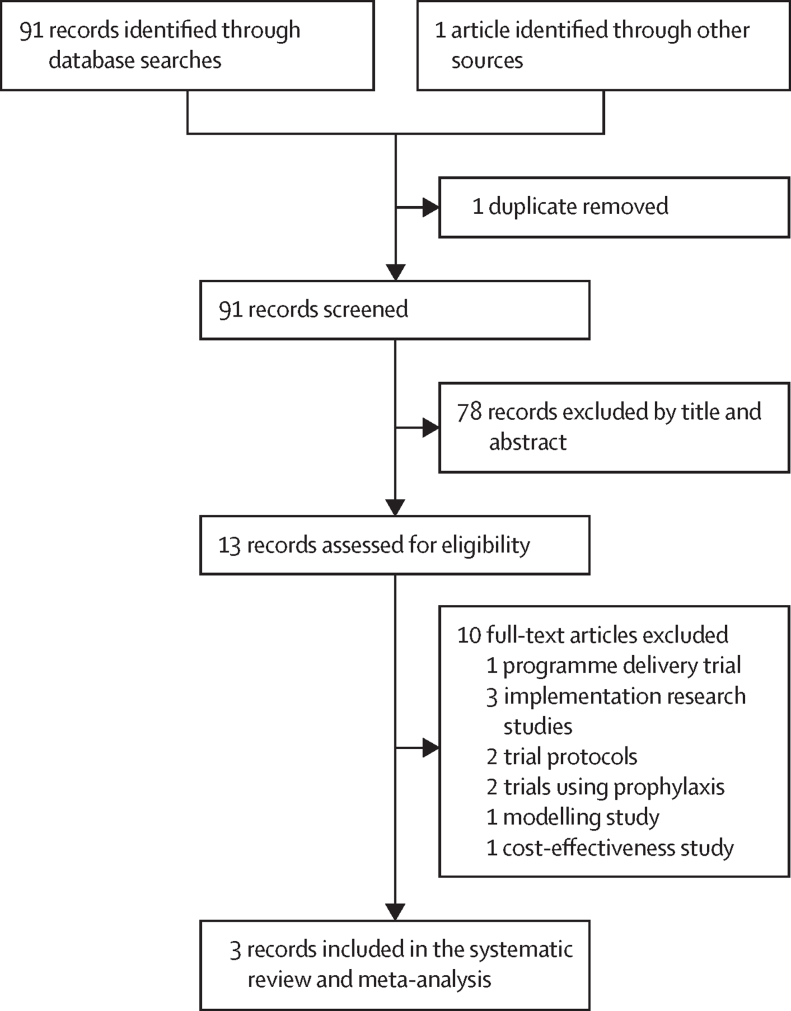
Study selection

Our database search identified 91 articles, with one additional article retrieved from the reference list of one of the identified articles. After removing duplicates and screening titles and abstracts, 13 full-text articles were evaluated, including five randomised controlled trials evaluating post-discharge chemoprevention in children with severe anaemia. Three trials were eligible (figure 1, table, appendix p 8). The trials were published between 2010 and 2020 and conducted at 18 sites in The Gambia,^8^ Malawi,^9^ Kenya, and Uganda.^10^ All three trials were double-blind and placebo-controlled and were scored as having a low risk of bias (appendix p 7). They included 3663 randomly assigned children with severe anaemia, 3507 (95·7%) of whom contributed to the modified intention-to-treat population. The two excluded trials used daily or weekly chemoprophylaxis post discharge instead of monthly administration of chemoprevention.^15^, ^16^

**Table:** Characteristics of included trials

	**Bojang and colleagues (2010)**^8^	**Phiri and colleagues (2012)**^9^	**Kwambai and colleagues (2020)**^10^
Countries	The Gambia	Malawi	Kenya and Uganda
Years of study	2003–04	2006–09	2016–18
Enrolled participants (PDMC:control)	1200 (600:600) randomly assigned while in hospital; 1085 (546:539) returned to receive their first course of PDMC or placebo 7 days after discharge and contributed to the analysis	1414 (706:708) randomly assigned while in hospital; 1373 (686:687) returned to receive their first course of PDMC or placebo 1 month after discharge and contributed to the analysis	1049 (524:525) randomly assigned 2 weeks post discharge; all contributed to the analysis
Design	Placebo-controlled	Placebo-controlled	Placebo-controlled
Health condition for admission	Severe anaemia (Hb <70 g/L) regardless of the presence of malaria parasites	Severe malarial anaemia (Hb <50 g/L and parenteral malarial treatment given)	Severe anaemia (Hb <50 g/L) regardless of the presence of malaria parasites
Initial case management in hospital provided to both study groups	Blood transfusion, if clinically indicated, intramuscular quinine or parenteral chloroquine followed by SP (for those with malaria)	Blood transfusion, parenteral quinine or artesunate, followed by AL	Blood transfusion, parenteral artesunate (for those with malaria), followed by AL (regardless of malaria)
Post-discharge intervention groups	Monthly SP (single-day dose) for the rest of the malaria transmission season, starting on day 7 post discharge; mean number of PDMC courses was 3·1 (range 1-6) and varied depending on the time in the transmission season when the participant was recruited	Monthly AL (3-day dose) at the end of week 4 and week 8 weeks post discharge	Monthly DHA–PiP (3-day dose) at the end of week 2 (around 14–15 days after discharge), week 6, and week 10 post discharge
Control group	Placebo SP	Placebo AL	Placebo DHA–PiP
Drug administration and adherence	All single-day doses given as directly observed therapy by study staff	The first daily doses of PDMC or placebo were provided in the community by study team members who visited each home in the morning for 3 days; the second daily dose was left with the caregiver to give in the evening; adherence was assessed the next morning	The first dose of each 3-day PDMC course was given as directly observed therapy, and the remaining two doses were left with the caregiver to administer to the child at home; daily telephone contact with caregivers and random home visits were used to verify adherence to the second and third dose of each 3-day course
Intervention period	Starting the day after the first PDMC course was given (day 7 post discharge) until 28 days inclusive after the last PDMC course or until the end of the malaria transmission season (Dec 31), whichever came last	8 weeks (week 5–12 post discharge), starting the day of the first 3-day course of PDMC (28–29 days post discharge) and ending 28 days inclusive after the first dose of the last course of PDMC or 12 weeks from enrolment (84 days inclusive), whichever came last	12 weeks (weeks 3–14 post discharge), starting the day of the first 3-day course of PDMC (14–15 days post discharge) and 28 days inclusive after the day of the first dose of the last course of PDMC or 14 weeks from enrolment (98 days inclusive), whichever came last
Post-intervention follow-up period	Approximately 4 months into the dry season, beginning the day after the end of the participant's intervention period until the day of the survey in May	13–26 weeks post discharge, beginning the day after the end of the participant's intervention period until the end of week 26 post discharge (day 182)	15–26 weeks post discharge, beginning the day after the end of the participant's intervention period until the end of the week 26 post discharge (day 182)
Key inclusion criteria for age and bodyweight	Age 3 months to 9 years	Age 4–59 months	Age <5 years, bodyweight ≥5·0 kg
Available data	IPD for all endpoints except mortality, which was available as aggregated data (numerator and denominator) by group and intervention period; the date of death was not available for all participants	IPD for all endpoints	IPD for all endpoints
Follow-up time in days, median (IQR)			
Intervention	66 (56–101)	57 (55–62)	85 (84–90)
Post intervention	Not applicable*	95 (90–103)	81 (75–83)
Overall	Not applicable*	152 (147–160)	168 (168–168)

PDMC=post-discharge malaria chemoprevention. Hb=haemoglobin concentration. SP=sulfadoxine–pyrimethamine. AL=artemether–lumefantrine. DHA–PiP=dihydroartemisinin–piperaquine. IPD=individual patient data.

* All children in this study were seen again in May the following calendar year, approximately 5 months into the dry season, for the assessment of vital status.

The first trial involved 1085 children with severe anaemia (haemoglobin <70 g/L), including children with non-malarial severe anaemia, and was conducted in 2003–04 in The Gambia with seasonal malaria transmission.^8^ This trial used monthly supervised treatment courses with sulfadoxine–pyrimethamine or placebo provided until the end of the malaria transmission season (July–December inclusive; mean number of courses 3·1, range 1–6). At the time of the study, high-grade sulfadoxine–pyrimethamine resistance was absent^8^ and seasonal malaria chemoprevention had not yet been introduced.

The second trial, conducted in 2006–09 in areas of Malawi with perennial malaria transmission, involved 1373 children with severe malarial anaemia (haemoglobin <50 g/L).^9^ Children in both study groups received artemether–lumefantrine at discharge and then artemether–lumefantrine or placebo at 4 weeks and 8 weeks post discharge, providing about 11–12 weeks of protection. Each day, the first artemether–lumefantrine dose was given by study staff at home, and the second was left with the caregiver to administer to the child later that day. Adherence was assessed the next morning by home visits. Children were followed up for 26 weeks.

The third trial involved 1049 children with severe anaemia (haemoglobin <50 g/L), including severe non-malarial anaemia, and was conducted in 2016–18 in areas of Uganda and Kenya with perennial malaria transmission.^10^ All children in both study groups received presumptive courses of artemether–lumefantrine at discharge and then either monthly dihydroartemisinin–piperaquine or placebo at the end of weeks 2, 6, and 10 post discharge, providing a total of 14 weeks of prophylaxis. The first dose of each 3-day post-discharge malaria chemoprevention was given by study staff at home. The second and third daily doses were left with the caregiver to administer to the child. Daily telephone calls and random spot checks at home were used to assess adherence. Children were followed up for 26 weeks (table, appendix p 8).

**Figure 2:**
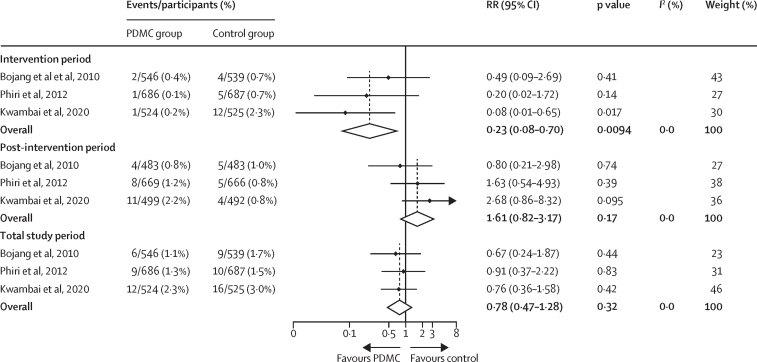
Effect of PDMC on mortality by study period The studies by Phiri and colleagues^9^ and Kwambai and colleagues^10^ were based on individual patient data. The study by Bojang and colleagues^8^ was based on aggregated data obtained from the source publication. Measure of effect modification by intervention period on the additive scale: relative excess risk due to interaction 1·26 (95% CI 0·62–1·90), p=0·0001. Measure of effect modification on the multiplicative scale: ratio of RRs 8·60 (95% CI 2·45–30·15), p=0·0008. PDMC=post-discharge malaria chemoprevention. RR=risk ratio.

During the intervention period, children in the post-discharge malaria chemoprevention groups were less likely to die post discharge than those in the placebo groups (RR 0·23 [95% CI 0·08–0·70], p=0·0094, *I*^2^=0%, figure 2), corresponding to a protective efficacy of 77% (95% CI 30–92) and an absolute risk reduction of 1·2% (95% CI 0·5–1·4) from an assumed control risk of 1·5% in the control group (appendix p 6) to 0·3% in the post-discharge malaria chemoprevention group. The NNT to avert one death during the intervention period was 113 (95% CI 66–396). The protective effect was only evident during the intervention period and was not sustained during the post-intervention period (RR 1·61 [0·82–3·17], p=0·17, *I*^2^=0%). There was no evidence of a cumulative beneficial effect on mortality at the end of the follow-up period (RR 0·78 [0·47–1·28], p=0·32, *I*^2^=0%). The difference in effect between the two periods was statistically significant (RERI 1·26 [95% CI 0·62–1·90], p=0·0001).

**Figure 3:**
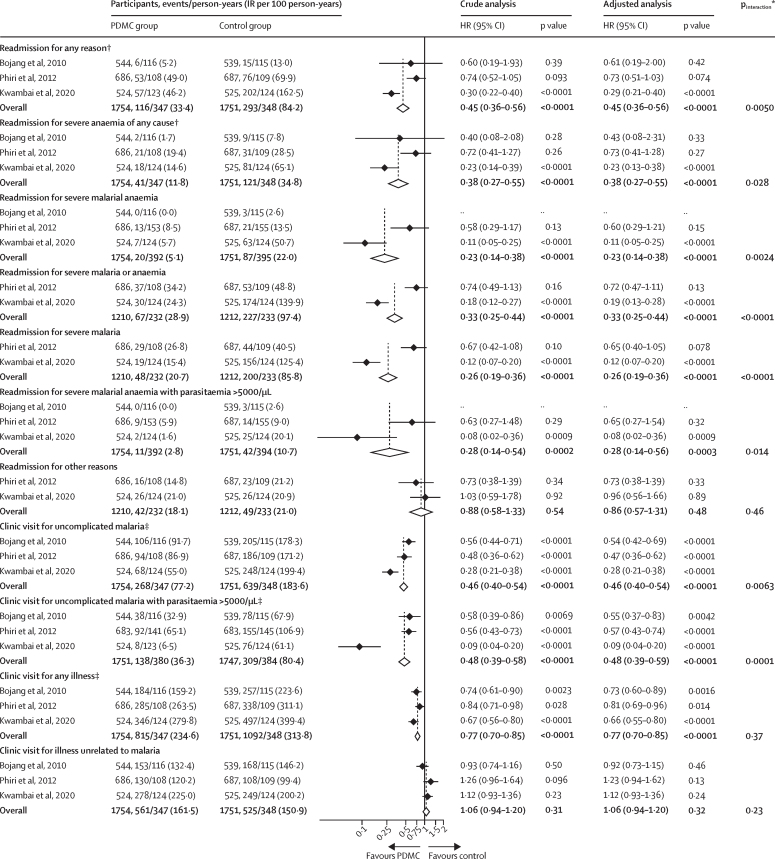
Effect of PDMC on readmission and clinic visits during the intervention period The source studies contributing to this analysis were by Bojang and colleagues,^8^ Phiri and colleagues,^9^ and Kwambai and colleagues.^10^ The adjusted models included five additional covariables: previous hospitalisation, bednet use, cubic of age in months, dose in mg/kg, and sex. HR=hazard ratio. IR=incidence rate. PDMC=post-discharge malaria chemoprevention. *p value for differences in treatment effect by study assessed by the ANOVA function on the full and reduced model. †The numbers of readmissions for any reason and readmissions for severe anaemia in the study by Bojang and colleagues^8^ are higher than the number reported in the source publication because in the current analysis, children with severe anaemia (haemoglobin <50 g/L) who were treated as outpatients (two children in the PDMC group and nine in the placebo group) were included under the readmission outcomes for consistency with the other two trials. ‡Proportional hazards assumption violated (see appendix p 10 for results by negative binomial regression).

Children in the post-discharge malaria chemoprevention groups had fewer all-cause readmissions than those in the control group during the intervention period (HR 0·45 [95% CI 0·36–0·56], p<0·0001), corresponding to a protective efficacy of 55% (95% CI 44–64; figure 3). The NNT to prevent one readmission was ten (95% CI 7–17). Overall, 101 (5·8%) of 1756 children in the PDMC groups were readmitted at least once, compared with 217 (12·4%) of 1751 in the placebo groups (appendix p 9). The effect was seen across events, with a 57% reduction in the first readmission (HR 0·43 [0·34–0·54], p<0·0001) and a 78% reduction in the second readmission (HR=0·22 [0·12–0·39], p<0·0001; appendix pp 9, 15) in the post-discharge malaria chemoprevention groups compared with the placebo groups. Similar results were seen in sensitivity analyses using count models or alternative Cox regression models (appendix p 14). The IRR obtained by negative binomial regression was 0·42 (95% CI 0·33–0·53, p<0·0001; appendix p 10).

Readmissions due to severe malaria were reduced by 74% (two studies, HR 0·26 [95% CI 0·19–0·36], p<0·0001; figure 3) in the post-discharge malaria chemoprevention groups compared with the placebo groups, readmissions due to severe anaemia by 62% (three studies, 0·38 [0·27–0·55], p<0·0001), and readmissions due to severe malarial anaemia by 77% (two studies, 0·23 [0·14–0·38], p<0·0001). Post-discharge malaria chemoprevention was also associated with a 23% reduction in non-severe all-cause sick-child clinic visits (0·77 [0·70–0·85], p<0·0001) and a 54% reduction in clinic visits for uncomplicated clinical malaria (0·46 [0·40–0·54], p<0·0001). There was no evidence for clinically relevant reductions in clinic visits for illnesses unrelated to malaria (1·06 [0·94–1·20], p=0·31). For some of these endpoints, the proportional hazard assumption was violated. Results are therefore also provided as IRRs (95% CIs) obtained by negative binomial regression (appendix pp 10–11).

**Figure 4:**
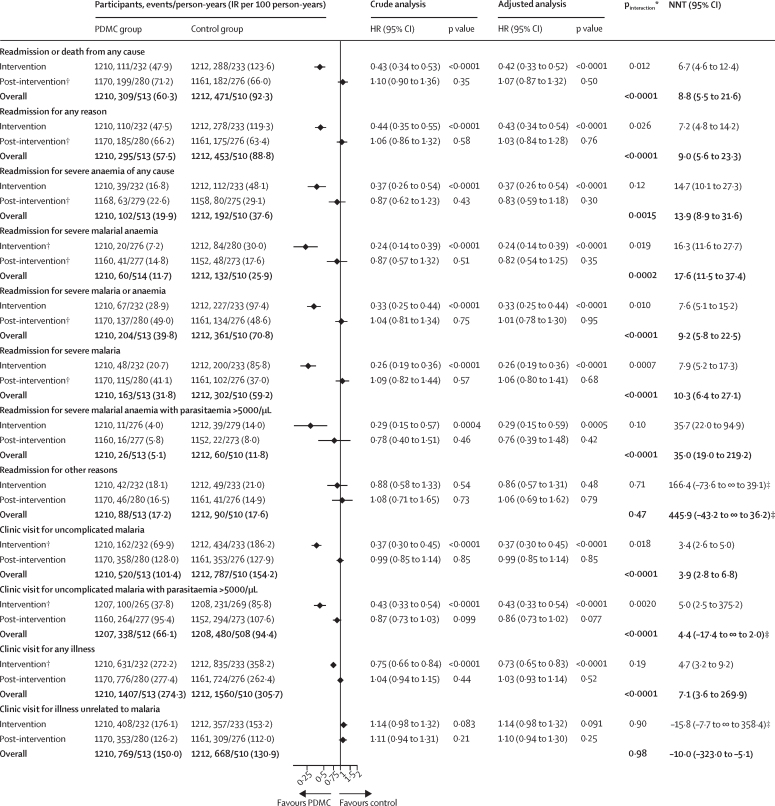
Outcomes by intervention period for other secondary outcomes (two trials) The two source studies contributing to this analysis were by Phiri and colleagues^9^ and Kwambai and colleagues.^10^ HR=hazard ratio. IR=incidence rate. PDMC=post-discharge malaria chemoprevention. NNH=number needed to harm. NNT=number needed to treat. The p values for the multiplicative (interaction HR) and additive interaction terms (relative excess risk due to interaction) represent the difference in treatment effect between the intervention and post-intervention periods. *p value for the additive interaction (top row) and multiplicative interaction (bottom row). †Proportional hazards assumption violated (see appendix p 11 for results by negative binomial regression). ‡The left CI illustrates NNH and the right CI illustrates NNT; the ∞ symbol illustrates that the NNH or NNT includes infinity.^17^

The assessment of the treatment effect by intervention period for the secondary outcomes could only be assessed in the trials by Phiri and colleagues and Kwambai and colleagues, because details for the post-intervention period were not available for the study by Bojang and colleagues beyond mortality. The composite of readmissions or death from any cause was 57% lower in the post-discharge malaria chemoprevention groups than the control groups during the intervention period (HR 0·43 [95% CI 0·34–0·53], p<0·0001; figure 4), but this was not seen during the post-intervention period (1·10 [0·90–1·36], p=0·35). The overall cumulative effect by 6 months post discharge remained clinically significant (NNT=9 [95% CI 6–22]). Similar findings were seen for all-cause readmissions and cause-specific readmission due to severe malaria, severe anaemia of any cause, or severe malarial anaemia. There was no evidence for clinically relevant reductions during the post-intervention period in the incidence of clinic visits for any illness, clinic visits for uncomplicated malaria, clinical malaria-associated high-density parasitaemia, or illness unrelated to malaria, yet marked reductions were seen during the intervention period. Differences in treatment effect between the intervention and post-intervention periods were evident for all of these outcomes except readmissions or clinic visits unrelated to malaria (figure 4).

**Figure 5:**
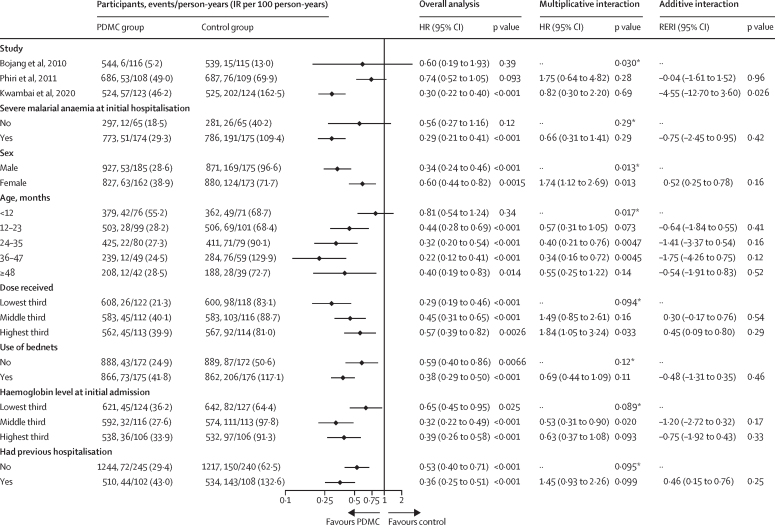
Subgroup analysis of readmissions for any reason The source studies contributing to this analysis were by Bojang and colleagues,^8^ Phiri and colleagues,^9^ and Kwambai and colleagues.^10^ The subgroup analysis for severe malarial anaemia at initial hospitalisation includes data from Bojang and colleagues and Kwambai and colleagues only. The p values for the interaction term represent the difference in treatment effect between the subgroups. IR=incidence rate. PDMC=post-discharge malaria chemoprevention. HR=hazard ratio. Interaction HR=ratio of hazard ratios (multiplicative interaction). RERI=relative excess risk due to interaction. *p value for the overall differences in treatment effect by subgroup assessed by the ANOVA function on the full and reduced model.

Further analyses were conducted to explore differences by subgroup in treatment effect during the intervention period for all-cause readmissions, the key secondary outcome for which IPD were available from all three trials (figure 5). The protective efficacy was greater in the study using dihydroartemisinin–piperaquine for post-discharge malaria chemoprevention (70%)^10^ than in the studies using sulfadoxine–pyrimethamine (40%)^8^ or artemether–lumefantrine (26%;^9^ p_interaction_=0·030). The protective efficacy was also greater in boys than in girls (66% *vs* 40%, p_interaction_=0·013) and smaller in infants (<12 months) than in older children (19% *vs* 56–78%; p_interaction_=0·017). Additionally, post-discharge malaria chemoprevention was protective in children with severe malarial anaemia on admission (71%) and those admitted with severe anaemia from other causes (44%; two studies, p_interaction_=0·29). The protective effect was also seen in both bednet users and non-users (62% *vs* 41%; p_interaction_=0·12), in children with and without a history of previous hospitalisation (64% *vs* 47%; p_interaction_=0·095), and in any of the tercile groups for haemoglobin level at the initial admission (p_interaction_=0·089).

Overall, all three drugs were well tolerated as monthly chemoprevention. No severe cutaneous reactions suggestive of Stevens-Johnson syndrome were seen with sulfadoxine–pyrimethamine. Minor symptoms recorded during the 30 days after the administration of each treatment were similar in the sulfadoxine–pyrimethamine and placebo groups.^8^ No drug-related serious adverse events were reported with monthly artemether–lumefantrine.^9^ Dihydroartemisinin–piperaquine (n=33) was associated with an 18·6 ms (95% CI 15·6 to 21·8) increase in the QTcF interval (Fridericia's method) after the third dose of each course (all asymptomatic), whereas placebo (n=33) was not (–1·8 ms [–5·3 to –1·7]; p<0·0001). The mean QTcF prolongation decreased with each subsequent course and was lower after the third compared with the first course of post-discharge malaria chemoprevention with dihydroartemisinin–piperaquine (p=0·022). None of the 33 children in the dihydroartemisinin–piperaquine group experienced QTcF values greater than 480 ms. The proportion of participants who vomited the study medication at least once within 60 min after drug intake was higher with dihydroartemisinin–piperaquine (12·4%) than with placebo (3·8%), but this did not result in any children having to stop the study medication.^10^

## Discussion

This is the first meta-analysis of monthly malaria chemoprevention trials for the efficacy of post-discharge care of African children who survived hospital admission for severe anaemia. The combined data show that approximately 3 months of post-discharge malaria chemoprevention could prevent three out of every four post-discharge deaths and more than 50% of all-cause hospital readmissions. The NNT to avert one death was 113, and to avert one readmission was 10. Post-discharge malaria chemoprevention also halved the number of clinic visits due to uncomplicated malaria. The direction of the effect was consistent across all three trials and was observed in children admitted with malaria-associated severe anaemia and severe anaemia due to other causes, and in both bednet users and non-users. Reduced readmissions were primarily due to fewer admissions for severe malaria or severe malarial anaemia. These results suggest that post-discharge malaria chemoprevention is an effective intervention that could have a high impact per child treated in preventing death or readmissions post discharge in areas with intense malaria transmission in Africa.

The protective effect was restricted to the intervention period and was not sustained after the direct pharmacodynamic effect of the drugs had waned. The studies by Kwambai and colleagues^10^ and Phiri and colleagues,^9^ which followed up children for 6 months, showed that after protective drug levels had waned, readmission and outpatient clinic visit rates increased to similar levels as in the control groups. There was some indication that all-cause mortality during the post-intervention period was higher in the post-discharge malaria chemoprevention group (RR 1·61 [95% CI 0·82–3*·*17], p=0·17), consistent with an increased risk of uncomplicated clinical malaria seen in previous seasonal malaria chemoprevention studies in children.^18^, ^19^ However, in the current study, this finding is unlikely to reflect a delayed acquisition of malarial immunity because there was no evidence post intervention of an increase in uncomplicated (HR 0·99 [95% CI 0·85–1·14], p=0·85) or severe malaria (1·09 [0·82–1·44], p=0·57) in the post-discharge malaria chemoprevention groups compared with the placebo groups. It could reflect a built-in selection bias because of the differential loss of the most susceptible children between study groups during the intervention period, as was suggested in the trial by Kwambai and colleagues.^10^ Overall, the initial 77% reduction in mortality during the intervention period outweighs the 61% increase during the post-intervention period; thus the cumulative effect by the end of the follow-up was still in favour of post-discharge malaria chemoprevention in all three studies and clinically relevant, although the confidence intervals were wide (RR 0·78 [95% CI 0·47–1·28], p=0·32).

Interventions that protect for longer than 3–4 months might further boost the effect of post-discharge malaria chemoprevention. In the study by Kwambai and colleagues, which provided the longest protection (14 weeks), nearly one in five (188 [18·9%] of 991) surviving children followed up for 6 months were either readmitted or died in the 12 weeks after the protective drug levels had waned.^10^ Longer post-discharge malaria chemoprevention courses are one option, but the study by Bojang and colleagues, which provided monthly post-discharge malaria chemoprevention with sulfadoxine–pyrimethamine for the rest of the transmission season, showed that adherence was initially high but decreased progressively at subsequent courses in participants who were scheduled to take more than three courses.^8^ This finding suggests that three courses, spaced monthly after discharge, could provide the right pragmatic balance. Longer courses could be considered when delivery platforms are created to deliver chemoprevention in communities, such as for perennial malaria chemoprevention (an extension of intermittent preventive treatment [IPT] in infants), and similar to the experience with monthly seasonal malaria chemoprevention, which is now given up to five times in some parts of west Africa. Another option to prolong the duration of protection is malaria monoclonal antibody therapy, which can potentially provide at least 6 months of protection against malaria.^20^ Ideally, children should also receive a long-lasting insecticide-treated bednet at discharge.

Subgroup analysis suggested that post-discharge malaria chemoprevention should not just be restricted to children with severe malarial anaemia, which comprised 62% of initial admissions with severe anaemia in The Gambia and 85% in Uganda and Kenya, but should also be given to children with non-malarial severe anaemia. Children initially admitted with non-malarial causes of severe anaemia are likely to have more complex, multifactorial aetiologies than children with severe malarial anaemia.^21^ Nevertheless, post-discharge malaria chemoprevention still resulted in a 44% reduction in all-cause readmissions compared with 71% in children with severe malarial anaemia. Providing post-discharge malaria chemoprevention to all children with severe anaemia in highly malaria-endemic areas, regardless of whether they have malaria during the initial hospitalisation, could be a pragmatic solution, provided they are not already scheduled to receive malaria chemoprevention for other reasons such as seasonal malaria chemoprevention or sickle cell disease.

In settings with high-grade sulfadoxine–pyrimethamine resistance, as in most of east and southern Africa, dihydroartemisinin–piperaquine is currently the most suitable candidate for chemoprevention and the most effective of the three drugs included in these trials. There is now considerable evidence corroborating the safety of monthly prophylaxis with dihydroartemisinin–piperaquine from studies in pregnant women,^22^, ^23^, ^24^, ^25^, ^26^, ^27^, ^28^ adults,^29^ children aged 6–24 months,^30^ and as seasonal malaria chemoprevention.^31^, ^32^, ^33^ In the post-discharge malaria chemoprevention trial by Kwambai and colleagues,^10^ which included nested cardiac monitoring, monthly courses of dihydroartemisinin–piperaquine were well tolerated. No serious adverse events attributable to the study drug were observed. As expected, asymptomatic QTc interval prolongation on the electrocardiogram was higher with dihydroartemisinin–piperaquine than with placebo, but no arrhythmias or clinical adverse events were observed, and none of the QTc intervals exceeded 480 ms. Furthermore, QTc prolongation decreased with each monthly course, consistent with previous trials in pregnancy.^25^, ^27^, ^28^, ^34^ Up to 18 monthly treatment courses of dihydroartemisinin–piperaquine have been safely given to children younger than 2 years who received monthly courses from age 6 months onwards.^30^

Monthly artemether–lumefantrine and sulfadoxine–pyrimethamine were also well tolerated. Artemether–lumefantrine provided the shortest post-treatment prophylaxis, evidenced by the sharp increase in clinical malaria cases seen 21 days after each course.^9^ It might not be ideal as monthly chemoprevention, especially in settings where artemether–lumefantrine is also used as first-line treatment for malaria case management. In west Africa, where high-grade sulfadoxine–pyrimethamine resistance is rare, the combination of sulfadoxine–pyrimethamine plus amodiaquine, widely used for seasonal malaria chemoprevention, could be an alternative in areas where seasonal malaria chemoprevention is not being implemented.

These results are consistent with a trial (excluded from our meta-analysis) from the 1990s using weekly prophylaxis with pyrimethamine–dapsone post discharge, which found a 78% reduction in readmissions and a 60% reduction in clinical malaria.^15^ By contrast, another excluded study using 3 months of bacterial and malaria prophylaxis with daily co-trimoxazole post discharge did not find any effect on mortality or all-cause readmissions, but severe adverse events with malaria were reduced by 23%.^16^ However, this study was done in areas with high-grade antifolate resistance, which probably affected the antimalarial efficacy of co-trimoxazole.^30^

With large-scale drug administration, there is always a concern about the spread of drug resistance. Although none of the studies were powered to address this, the fraction of the population targeted by post-discharge malaria chemoprevention and the corresponding selective drug pressure on the parasite population is much smaller than with seasonal malaria chemoprevention, IPT in pregnancy, perennial malaria chemoprevention (previously IPT in infants), or IPT in schoolchildren, which each include all members of a target population regardless of health status.^35^

Health services research has shown that post-discharge malaria chemoprevention is potentially cost saving^36^ and highly acceptable to caregivers and community health workers.^37^, ^38^ Unlike seasonal malaria chemoprevention, IPT in pregnancy, or perennial malaria chemoprevention, no health-care delivery platform is currently designated to support delivery of post-discharge malaria chemoprevention. It is directed at a small, seriously ill fraction of the population already connected to the health-care system (ie, those who are hospitalised).^39^ This in-hospital period provides an opportunity to engage with the caregivers and provide clear and context-specific health education messages to ensure adequate coverage of all post-discharge malaria chemoprevention courses under programmatic conditions. A delivery mechanism trial from 2021 showed that providing all three post-discharge courses to the caregivers at discharge achieved better coverage than facility-based delivery that required caregivers to return to the facility to collect their child's next course.^40^ This could be combined with mobile telephone text reminders or home visits by community health workers.^40^

WHO recommends post-discharge malaria chemoprevention for moderate to high perennial malaria transmission settings, defined as areas with a *Plasmodium falciparum* parasite prevalence greater than 10% in children aged 2–10 years or an annual parasite incidence greater than 250 per 1000 population.^7^ A mathematical model of the projected impact of post-discharge malaria chemoprevention across malaria-endemic African countries suggested that if all hospitalised children aged 0–5 years with severe anaemia were given post-discharge malaria chemoprevention in these areas, 38 600 readmissions (range 16 900–88 400) and 2176 deaths (1078–4315) could be prevented annually.^35^ The impact would be greatest in countries with higher transmission intensities. In areas with a *P falciparum* prevalence greater than 10% in children aged 2–10 years, an estimated 4·8 children would need to be given post-discharge malaria chemoprevention to prevent one readmission, and 112 children to prevent one death, consistent with the findings in this meta-analysis. In the two highest-burden countries, Nigeria and the Democratic Republic of the Congo, only 3·1 and 2·9 children need to be given post-discharge malaria chemoprevention to prevent a hospitalised episode, and 55 and 53 to prevent one death, respectively.^35^

This meta-analysis has several limitations. First, only three trials were included, and each trial used a different drug and slightly different regimen. The small number of trials limited our ability to assess heterogeneity and its sources and publication bias. Second, mortality data in the study by Bojang and colleagues were only available as aggregated data. Third, the mortality analysis is subject to sparse data bias^41^ due to the small number of deaths, particularly during the intervention period in the post-discharge malaria chemoprevention groups, resulting in unrealistically large HR estimates (eg, 0·08) and confidence limits (eg, 0·01) in some studies,^10^ which carry over into the pooled HR estimate. Fourth, the period-specific analysis is limited by the inherent built-in selection bias because of the differential depletion of the most susceptible children during the intervention period.^42^ Other limitations include scarce diagnostic data for the non-malaria causes of post-discharge readmissions or deaths. Furthermore, the absolute difference in mortality might have been underestimated, resulting in a higher NNT estimate to avert one death, because the mortality rate in the control group in all three trials was lower than previously observed in the post-discharge community at large.^4^ This finding might reflect enhanced access to standard care as a result of participating in a trial, including the early diagnosis of events requiring readmission.

Future research should focus on post-discharge malaria chemoprevention delivery methods or interventions that can prolong the protection duration beyond 4 months, and other hospitalised groups at high risk, such as children admitted with severe malaria without severe anaemia, or children with severe acute malnutrition. Other interventions could also be considered, such as anthelmintics or those that address additional nutritional factors or recurrent bacterial infections. However, they may be less generalisable and require tailoring to local modifiable risk factors.

In conclusion, this meta-analysis confirms the high risk of malaria-associated readmissions and death post discharge and supports the WHO recommendation for monthly malaria chemoprevention with long-acting antimalarials as a valuable new strategy for the post-discharge care of children with severe anaemia in areas with moderate to high perennial malaria transmission.

## Data sharing

Individual patient data for studies contributing to this analysis are available from the authors of the three source studies.

## Declaration of interests

We declare no competing interests.
