# Supplementary material for: Post-discharge malaria chemoprevention in children admitted with severe anaemia in malaria-endemic settings in Africa: a systematic review and individual patient data meta-analysis of randomised controlled trials
Source: Lancet Glob Health. 2023 Dec 12;12(1):e33–44. doi: 10.1016/S2214-109X(23)00492-8 (PMC10733130; doi:10.1016/S2214-109X(23)00492-8)
Supplement: Supplementary appendix [file mmc1.pdf]

THE LANCET
Global Health

Supplementary appendix

This appendix formed part of the original submission and has been peer reviewed. We post it as supplied by the authors.

Supplement to: Phiri KS, Khairallah C, Kwambai TK, et al. Post-discharge malaria chemoprevention in children admitted with severe anaemia in malaria-endemic settings in Africa: a systematic review and individual patient data meta-analysis of randomised controlled trials. *Lancet Glob Health* 2024; **12:** e33–44.

Supplementary appendix

Supplement to: Phiri et al., Post-discharge Malaria Chemoprevention in Children Admitted with Severe Anaemia in Malaria-Endemic Settings in Africa: A systematic review and Individual Patient Data meta-analysis of randomised controlled trials

Table of Contents

Supplemental methods 3
Supplement 1: Search strategy 3
Supplement 2: Quality and risk of bias assessment of trials 3
Supplement 3: Definition of outcomes 3
Primary outcome 3
Secondary outcomes (by intervention period and overall) 3
Supplement 4: Periods of assessment 3
Supplement 5: Statistical analysis 4
Statistical models 4
Duration of effect; comparison between intervention and post-intervention periods 5
Number-needed-to-treat (NNT) 6
Heterogeneity 6
Supplemental tables 7
Table S1: Cochrane collaboration tool for quality assessment of randomised controlled trials 7
Table S2: Narrative summary of included trials 8
Table S3: Number of all-cause readmissions by study period and study 9
Table S4: Negative binomial regression for secondary outcomes related to Figure 3 in the main text (three trials)[4-6] 10
Table S5: Negative binomial regression by intervention period for other secondary outcomes related to Figure 4 in the main text (two trials)[5,6] 11
Table S6: Impact of different transformations of continuous covariates on the overall model fit and the adjusted effect size estimate for the effect of PDMC on all-cause readmissions 12
Table S7: Number, proportion and reasons for censoring by endpoint 13
Supplemental figures 14
Figure S1: Sensitivity analysis of all-cause readmissions using alternative models 14
Figure S2: Kaplan Meier curves and treatment effect on time to first and second all-cause readmission using standard Cox regression 15

Figure S3: Interaction by study for other secondary outcomes (three trials) 16
Figure S4: Interaction by study period for other secondary outcomes (two trials) 17
Supplemental references 18

Supplemental methods

Supplement 1: Search strategy

We identified eligible studies by performing a literature search using a combination of search terms in PubMed, SCOPUS, EMBASE, Web of Science, Cochrane CENTRAL, and the WHO's clinical trial registry and by searching in Google and Google Scholar. Randomised controlled trials were eligible if they were conducted in a malaria-endemic area of Africa[1] among children <15 years of age recently discharged after hospitalisation for severe anaemia and compared monthly malaria chemoprevention regimens after discharge against placebo or the current standard of post-discharge care. Trials using daily or weekly malaria prophylaxis were not eligible. The search was conducted in English but without language restrictions.

The following search terms were used in PubMed: (child OR childhood OR infant OR pediatric OR paediatric) AND (malaria OR plasmodium) AND ("severe anaemia" OR "severe anemia" OR transfusion) AND (recurrence OR discharge OR postdischarge OR post-discharge).

The authors of eligible trials were approached for pseudonymised individual participant datasets. Datasets were standardised for subsequent inclusion in the master database used for analysis.

Supplement 2: Quality and risk of bias assessment of trials

The risk of bias assessment for each included trial was conducted by two investigators (TKK and FtK) using version 2 of the Cochrane risk-of-bias tool for randomised trials (RoB 2).[2,3] RoB2 is structured into a fixed set of domains of bias, focusing on different aspects of trial design, conduct and reporting. A judgement about the risk of bias arising from each domain is proposed by an algorithm and can be overwritten by the authors with justification. Judgements can be a 'low' or 'high' risk of bias or expressed as 'some concerns'. Where disagreement occurred, a joint review of the study was conducted until agreement was reached by consensus. Studies were not excluded *a priori* on the basis of their quality score.

Supplement 3: Definition of outcomes

Primary outcome

All-cause death during the intervention period

Secondary outcomes (by intervention period and overall)

- All-cause deaths during the post-intervention follow-up period and overall
- All-cause readmissions
- All-cause death or readmissions (composite)
- Cause-specific readmissions
- Severe malarial anaemia readmissions (≥5000 parasites/µL and haemoglobin level<5g/dL)
- All-cause non-severe sick-child visits
- Uncomplicated clinical malaria, defined according to the data reported in the source studies as a non-severe sick-child clinic visit resulting in receipt of oral antimalarials for confirmed or presumptive malaria infections.
- Uncomplicated clinical malaria with high-density parasitaemia (≥5000 parasites/µL)
- Non-malarial non-severe sick-child visits

Supplement 4: Periods of assessment

The analysis was stratified *a priori* by the PDMC-intervention period (primary analysis) and a post-intervention period (evaluated in those who survived the intervention period), and 'overall', defined as the cumulative effect across both periods pooled. This was done to provide independent estimates of the direct effect of the intervention (PDMC-intervention period) and to assess whether any rebound or delayed episodes occurred during the post-intervention period when the direct pharmacological

protective effect of the antimalarial drugs had waned. It also allowed us to determine the overall cumulative effect at the end of the post-intervention follow-up.

The intervention period was defined as the period starting from the first dose of the first course of PDMC until four weeks (28 days) after the first dose of the last scheduled course of PDMC. The timing of the first course of PDMC varied by the source study from approximately 7,[4] 14[5] or 28 days[6] post-discharge. This period is henceforth referred to as the intervention period. In the study in the Gambia, the intervention was provided for the duration of the malaria transmission season (July to December inclusive).[4] For the purpose of this analysis, the intervention period was defined as ending 28 days after the last course of PDMC given in the malaria transmission season. For example, if the last course of PDMC was given on December 31, then the intervention period for that child ended on January 28 of the next year. If the last course was given on December 21, the intervention period ended on January 19. If the last course was given on December 03 or earlier, the intervention period ended on December 31.

The post-intervention period was defined as the period starting the day after the completion of the intervention period (see above) up to 26 weeks post-discharge (day 182) in the trials in Malawi,[6] Kenya and Uganda,[5] or until the assessment approximately five months into the dry season in the trial from the Gambia (the month of May).[4]

Supplement 5: Statistical analysis

Statistical models

Mortality data were available as IPD for 2 studies[5,6] and as aggregated data for 1 study in The Gambia.[4] Time-to-death was not available for this study in The Gambia, only count data on the total number of deaths by intervention period.[4] The impact on mortality data was therefore analysed using fixed-effects two-stage meta-analyses of risk ratios. First, for each of the two studies with IPD.[5,6], the risk ratios for mortality were obtained by Generalised Linear Models (GLM) using the log-link function and a binomial distribution. These GLM models also included the stratification factors study site and the bodyweight category used at randomisation as fixed effect covariates. In the second stage, these were combined with the risk ratio obtained from the study in The Gambia, for which only aggregated data was available using the IPDmetan command in Stata. Results are described as risk ratios and 95% confidence intervals, and protective efficacy (PE), defined as PE=100%x[1-RR]. Because only three studies contributed to this analysis of mortality data, random-effects models were not considered because the between-study variance cannot be reliably estimated with a small number of studies.[7-10]

Recurrent time-to-event data were available from all three studies for all other efficacy outcomes. They were analysed using mixed effects Prentice-Williams-Peterson Total-Time (PWP-TT) models to obtain HRs.[11] PWP-TT models use a stratified Cox-based approach that relates the hazard function to preceding failure time history and allows the shape of the hazard function to depend on the number of preceding events.[12] For each participant, it considers the time since the start of the study (total time) and incorporates the number of previous events experienced by each participant. Thus, a single participant can contribute multiple times depending on the number of events.[13]

Results were analysed by study period (intervention period and post-intervention period). The definitions of the intervention and post-intervention study periods are given in the section "Supplement 4: Periods of assessment" (appendix p 3, above). In the database structure, there was one observation per event or time interval for each study period. The time of entry into the study period was defined as the day of the first dose of the first course of PDMC (intervention period) or 29 days after the first dose of the last scheduled course of PDMC (post-intervention period). For participants with no event, there was one observation per study period in the database covering the time from entry into the study period (intervention or post-intervention) until the end of that study

period, or until the time the patient was lost to follow-up or withdrew from the study during that study period (censoring). For participants with one event in a specific study period, there were two observations in the database for that study period. The first observation covered the time span from entry into the study period until the time of the event (end-time for that observation), and the second observation spanned the time from the first-and-only event (start-time for the second observation) to the end of follow-up of that study period, or until the time the patient was lost to follow-up or withdrew from the study during that study period (end-time for that observation). Similarly, for participants with two events in a specific period, there were three observations for that period. The first observation covered the time span from entry into the study period until the time of the first event in that study period (end-time for that observation), the second observation spanned the time from the first event (start-time for the second observation) until the second event in that study period (end time for the second observation) and the third observation spanned the time from the second event (start-time for the third observation) until the end of that study period or until the time the patient was lost to follow-up or withdrew from the study during that study period (end-time for the third observation). Similar approaches were used for patients with more than two events.

Because each study was conducted in multiple hospitals, the three studies included a total of 18 study 'sites' (Bojang et al. 5;[4] Phiri et al. 4,[6] Kwambai et al. 9[5]), which allowed the use of mixed-effects models. Each IPD model included study site as a random effect (with a random intercept for study site and patient nested within site) and the bodyweight category used at the time of randomisation as a fixed effect covariate to adjust for stratification factors. The adjusted models include five additional covariables available for all studies, including previous hospitalisation (yes/no), bednet use (yes/no), cubic of age (age^3), dose in mg per kg (terciles, categorical), and sex (male/female) because in previous studies there were found to be predictive of the rate of readmissions.[5,6] The optimal scale for continuous covariables was based on the Akaike Information Criterion (AIC) from various models to determine which scale resulted in the best model fit of the treatment effect on all-cause readmissions (appendix p 12). Results are described as hazard ratios and 95% confidence intervals and as protective efficacy (PE) defined as PE=100%x[1-RR], PE=100%x[1-IRR], or PE=100%x[1-HR], depending on the available data. To obtain the number needed to treat (NNT), incidence rate ratios (IRR) for readmissions for any reason were also calculated using negative binomial regression (appendix p 6).

Further sensitivity analyses to assess the robustness of the primary analysis were conducted using alternative time-to-event models and count models, including extended Cox regression with Prentice-Williams-Peterson Gap-Time and Andersen Gill models and count models using negative binomial regression and standard and zero-inflated Poisson regression (appendix p 14). The presence or absence of overdispersion in the count data was verified using "overdisp", a Stata module for the direct detection of overdispersion in Poisson and negative binomial regression Models.[14] Because significant overdispersion was present for many of the count outcomes, negative binomial regression was used as the main method for sensitivity analysis of endpoints for which the proportional hazard assumptions were violated.

Duration of effect; comparison between intervention and post-intervention periods

The analysis was stratified *a priori* by the PDMC-intervention period (starting from the first day of chemoprevention) (primary analysis) and a post-intervention period (evaluated in those who survived the intervention period), and 'overall', defined as the cumulative number of events averted by the end of the intervention period. This was done to provide independent estimates of the direct effect of the intervention (PDMC-intervention period) and to assess whether any rebound or delayed episodes occurred during the post-intervention period when the direct pharmacological protective effect of the

antimalarial drugs had waned. See appendix p 3 for definitions of the intervention period, post-intervention period and "overall" (the entire follow-up period).

The differences in treatment effect during the intervention period and the post-intervention period were explored using the multiplicative (the ratio of risk ratios [mortality] or ratio of hazard ratios [other ednpoints]) and additive interactions (the relative excess risk due to interaction [RERI], also referred to as the interaction contrast ratio (ICR), and the corresponding 95% confidence intervals andP-values ($P_{interaction}$).[15] These were obtained from one-stage mixed effects PWP-TT models for repeated events with treatment-covariate (study period) interactions. Similar one-stage analyses of treatment-covariate interactions were used for assessing differential responses to treatment by other subgroups, such as bednet use, age, gender and presence of malaria during the original hospital admission. For the two-stage aggregated data meta-analysis of mortality data, the multiplicative and additive (RERI) interactions for the treatment effect by study period, their 95% CIs and corresponding p-values were obtained using methods described by Richardson & Kaufman.[16]

The cumulative effect of PDMC for the overall treatment effect, i.e. over the entire follow-up period, could not be expressed as the hazard ratio because the proportional hazard assumption was not satisfied for most endpoints. Instead, negative binomial regression models were used to obtain estimates of the effect by intervention period and the cumulative effect at the end of the entire follow-up period, expressed as the IRR and NNT.

Number-needed-to-treat (NNT)

Mortality (IPD data available for 2 of 3 studies): Using a two-stage approach, the NNT to prevent one all-cause death was computed as NNT=1/RD where RD is the risk difference between PDMC and control arms by the end of the intervention period. First, the risk differences and corresponding 95% CIs for each of the two IPD studies were calculated from the GLM model for mortality (as described above) , using the margins command. In the second stage, these two RDs (95% CIs) were combined with the RD and 95% CIs obtained from the study in The Gambia (for which only aggregated mortality data were available) using the 'metan' command in Stata to obtain the pooled RD and 95% CIs. Because only three studies contributed, fixed-effects meta-analysis was used.[7-10] The lower and upper confidence limits of the NNT were also obtained by using the inverse of the pooled RD's upper and lower 95% confidence limit

Readmissions: The number-needed-to-treat (NNT) to prevent one readmission or clinic visit was computed as NNT=1/IRD, where IRD is the incidence rate difference calculated as the average difference of marginal incidence rates in the control and PDMC arms for that endpoint over the specific analysis period (intervention, post-intervention, and the entire period [overall]) obtained from negative binomial models with a random intercept for study site. The 95% confidence intervals of the NNT were calculated as the inverse for 95% CI of IRD estimated by the delta method using the "margins" and the user written "spost13" commands in Stata. ACRatex(1-IRR) represents the absolute rate reduction. Confidence intervals for NNTs derived from IRDs with 95% CIs that overlap with zero are expressed as NNT to harm (NNH) and NNT to benefit (NNT) with the infinity symbol (∞) in between to illustrate that the NNH or NNT include infinity (∞) as proposed by Altman et al.[19]

Heterogeneity

The extent of heterogeneity was measured using the I^2 statistic in the aggregated data meta-analysis of mortality data,[6] which is a measure of the proportion of total variability due to heterogeneity rather than chance, expressed as a percentage, with 0-40% representing no or little heterogeneity, 30-60% moderate heterogeneity, 50-90% substantial heterogeneity, and 75-100% considerable heterogeneity.[20]

Supplemental tables

Table S1: Cochrane collaboration tool for quality assessment of randomised controlled trials

	Random Sequence Generation	Allocation Concealment	Blinding of Participants and Personnel	Blinding of Outcome Assessment	Incomplete Outcome Data	Selective Reporting	Other Bias
Bojang, 2010[4]	+	+	+	+	+	+	+
Phiri, 2012[6]	+	+	+	+	+	+	+
Kwambai, 2020[5]	+	+	+	+	+	+	+

+	Low Risk of Bias	?	Unclear Risk of Bias	-	High Risk of Bias

Risk of bias assessment for included studies with the authors' judgements for each included trial. Adapted from the Revised Cochrane risk-of-bias tool for randomised trials (RoB 2).

Table S2: Narrative summary of included trials

Bojang et al., 2010[4]	The first trial was conducted in 2003-2004 in the Gambia among children with severe anaemia (including children with non-malarial severe anaemia), defined as a Hb<7/g/dL.[4] Out of 1,200 children randomised while in-hospital, 1,085 children were seen on day 7 after discharge for their first course of PDMC or placebo and contributed to the modified intention-to-treat analysis. This trial used monthly treatment courses with sulfadoxine-pyrimethamine (SP) or placebo provided until the end of the malaria transmission season (July-December inclusive). The number of courses varied depending on the time in the transmission season the participants were recruited. The average number of PDMC courses received was 3.1 (range 1 to 6). IPD were available for all-cause and cause-specific hospital readmission and outpatient visits during the intervention period (defined as the period ending 28 days after the last course of PDMC). Mortality data were available during both the intervention and post-intervention periods. This was assessed through home visits in January of each year to assess the impact during the intervention period and again in May (approximately five months into the dry season) to assess the impact during the post-intervention period. For this analysis, the post-intervention period was defined as the four months during the dry season, starting 29 days after the last course of PDMC. Mortality data were only available as aggregated data and the date of death, and therewith time-to-event, was not available for all children who had died. Results were therefore expressed as risk ratios. At the time of the study, the quintuple dhfr/dhps haplotype associated with high-grade sulfonamide resistance was absent in the Gambia,[4,21] and seasonal malaria chemoprevention (SMC) had not yet been introduced as national policy.
Phiri et al., 2012[6]	The second trial was conducted in 2006-2009 in four hospitals in southern Malawi, involving children with severe malarial anaemia (Hb<5g/dL).[6] Out of a total of 1,414 children randomised while in hopsital, 1,373 were seen 1 month after discharge for their first course of PDMC or placebo and contributed to the modified intention-to-treat analysis. Children in both arms received artemether-lumefantrine at discharge and then artemether-lumefantrine or placebo at 1 and 2 months post-discharge, providing about 11 to 12 weeks of protection.[13] Children were followed for six months. Results were available by the intervention period (1-3 months), post-intervention period (4-6 months) and overall (1-6 months post-discharge).
Kwambai et al., 2020[5]	The third trial was conducted in 2016-2018 in nine hospitals in Uganda and Kenya and involved children with severe anaemia (Hb<5g/dL), including severe non-malarial anaemia.[5] All children in both arms received presumptive courses of artemether-lumefantrine at discharge. 1,049 children were randomised approximatley 14 to 15 days post-discharge to receive either monthly dihydroartemisinin-piperaquine or placebo at the start of week 3, 7 and 11 weeks post-discharge, providing a total of 14 weeks of prophylaxis. All contributed to the modified intention-to-treat analysis. Children were followed for a total of 26 weeks, and results were available by the intervention period (2-14 weeks post-discharge), post-intervention period (15-26 weeks) and overall (2-26 weeks) for all outcomes.

Table S3: Number of all-cause readmissions by study period and study

	Intervention			Post-intervention		
	**PDMC**	**Control**	**Overall**	**PDMC**	**Control**	**Overall**
**Overall**	1754	1751	3505	1170	1161	2331
1 event only	87 (5.0)	166 (9.5)	253 (7.2)	133 (11.4)	116 (10.0)	249 (10.7)
2 events only	13 (0.7)	32 (1.8)	45 (1.3)	23 (2.0)	20 (1.7)	43 (1.8)
≥3 events	1 (0.1)	19 (1.1)	20 (0.6)	2 (0.2)	5 (0.4)	7 (0.3)
≥1 event	101 (5.8)	217 (12.4)	318 (9.1)	158 (13.5)	141 (12.1)	299 (12.8)
total events	116	293	409	185	175	360
**Bojang, 2010**	544	539	1083	na	na	na
1 event only	6 (1.1)	13 (13.0)	19 (1.8)	na	na	na
2 events only	0 (0.0)	1 (1.0)	1 (0.1)	na	na	na
≥3 events	0 (0.0)	0 (0.0)	0 (0.0)	na	na	na
≥1 event	6 (1.1)	14 (14.0)	20 (1.8)	na	na	na
total events	6	15	21			
**Phiri, 2012**	686	687	1373	671	669	1340
1 event only	44 (6.4)	55 (55.0)	99 (7.2)	52 (7.7)	53 (7.9)	105 (7.8)
2 events only	3 (0.4)	6 (6.0)	9 (0.7)	7 (1.0)	5 (0.7)	12 (0.9)
≥3 events	1 (0.1)	3 (3.0)	4 (0.3)	0 (0.0)	1 (0.1)	1 (0.1)
≥1 event	48 (7.0)	64 (64.0)	112 (8.2)	59 (8.8)	59 (8.8)	118 (8.8)
total events	53	76	129	66	70	136
**Kwambai, 2020**	524	525	1049	499	492	991
1 event only	37 (7.1)	98 (98.0)	135 (12.9)	81 (63.0)	63 (12.8)	144 (14.5)
2 events only	10 (1.9)	25 (25.0)	35 (3.3)	16 (15.0)	15 (3.0)	31 (3.1)
≥3 events	0 (0.0)	16 (16.0)	16 (1.5)	2 (4.0)	4 (0.8)	6 (0.6)
≥1 event	47 (9.0)	139 (139.0)	186 (17.7)	99 (19.8)	82 (16.7)	181 (18.3)
total events	57	202	259	119	105	224

Table S4: Negative binomial regression for secondary outcomes related to Figure 3 in the main text (three trials)[4-6]

Endpoint	Crude		Adjusted	
	IRR (95% CI)	p-value	IRR (95% CI)	p-value
Readmission for any reason*	0.419 (0.331, 0.529)	<0.0001	0.419 (0.332, 0.530)	<0.0001
Readmission for severe anaemia of any cause*	0.348 (0.239, 0.506)	<0.0001	0.353 (0.242, 0.513)	<0.0001
Readmission for severe malarial anaemia	0.229 (0.139, 0.376)	<0.0001	0.234 (0.142, 0.385)	<0.0001
Readmission for severe malaria or anaemia	0.312 (0.233, 0.418)	<0.0001	0.310 (0.232, 0.415)	<0.0001
Readmission for severe malaria	0.247 (0.178, 0.342)	<0.0001	0.247 (0.178, 0.343)	<0.0001
Readmission for severe malarial anaemia with parasitaemia >5000/µL	0.267 (0.135, 0.528)	0.0001	0.274 (0.138, 0.545)	0.0002
Readmission for other reasons	0.862 (0.558, 1.332)	0.50	0.840 (0.543, 1.301)	0.43
Clinic visit for uncomplicated malaria	0.415 (0.356, 0.484)	<0.0001	0.418 (0.358, 0.486)	<0.0001
Clinic visit for uncomplicated malaria with parasitaemia >5000/µL	0.448 (0.365, 0.550)	<0.0001	0.453 (0.369, 0.556)	<0.0001
Clinic visit for any illness	0.748 (0.678, 0.825)	<0.0001	0.751 (0.681, 0.827)	<0.0001
Clinic visit for illness unrelated to malaria	1.086 (0.953, 1.236)	0.22	1.090 (0.957, 1.240)	0.19

IRR=Incidence rate ratio obtained by negative binomial regression. The source studies contributing to this analysis included Bojang et al, 2010[4]; Phiri et al, 2012;[6] and Kwambai et al, 2020.[5]

Table S5: Negative binomial regression by intervention period for other secondary outcomes related to Figure 4 in the main text (two trials)[5,6]

Endpoint	Period	Crude IRR (95% CI)	Crude p-value	Adjusted IRR (95% CI)	Adjusted p-value
Readmission or death from any cause	Intervention	0.408 (0.321, 0.518)	<0.0001	0.401 (0.315, 0.511)	<0.0001
	Post-intervention	1.092 (0.880, 1.356)	0.42	1.064 (0.858, 1.319)	0.57
	Overall	0.686 (0.580, 0.812)	<0.0001	0.668 (0.564, 0.790)	<0.0001
Readmission for any reason*	Intervention	0.419 (0.329, 0.533)	<0.0001	0.414 (0.325, 0.527)	<0.0001
	Post-intervention	1.054 (0.845, 1.316)	0.64	1.028 (0.825, 1.280)	0.81
	Overall	0.681 (0.575, 0.807)	<0.0001	0.664 (0.560, 0.787)	<0.0001
Readmission for severe anaemia of any cause*	Intervention	0.359 (0.244, 0.530)	<0.0001	0.361 (0.245, 0.533)	<0.0001
	Post-intervention	0.804 (0.554, 1.169)	0.25	0.768 (0.529, 1.116)	0.17
	Overall	0.556 (0.420, 0.736)	<0.0001	0.547 (0.414, 0.723)	<0.0001
Readmission for severe malarial anaemia	Intervention	0.237 (0.144, 0.390)	<0.0001	0.242 (0.147, 0.399)	<0.0001
	Post-intervention	0.867 (0.563, 1.333)	0.51	0.843 (0.549, 1.295)	0.44
	Overall	0.469 (0.339, 0.648)	<0.0001	0.468 (0.339, 0.646)	<0.0001
Readmission for severe malaria or anaemia	Intervention	0.312 (0.233, 0.418)	<0.0001	0.310 (0.232, 0.415)	<0.0001
	Post-intervention	1.023 (0.791, 1.322)	0.86	0.996 (0.771, 1.286)	0.97
	Overall	0.597 (0.490, 0.728)	<0.0001	0.585 (0.480, 0.713)	<0.0001
Readmission for severe malaria	Intervention	0.247 (0.178, 0.342)	<0.0001	0.247 (0.178, 0.343)	<0.0001
	Post-intervention	1.119 (0.855, 1.466)	0.41	1.100 (0.842, 1.437)	0.48
	Overall	0.556 (0.452, 0.685)	<0.0001	0.549 (0.446, 0.676)	<0.0001
Readmission for severe malarial anaemia with parasitaemia >5000/µL	Intervention	0.288 (0.144, 0.574)	0.0004	0.294 (0.147, 0.590)	0.0006
	Post-intervention	0.765 (0.377, 1.553)	0.46	0.764 (0.373, 1.563)	0.46
	Overall	0.468 (0.287, 0.763)	0.0023	0.480 (0.294, 0.784)	0.0034
Readmission for other reasons	Intervention	0.862 (0.558, 1.332)	0.50	0.840 (0.543, 1.301)	0.43
	Post-intervention	1.107 (0.711, 1.724)	0.65	1.081 (0.694, 1.682)	0.73
	Overall	0.972 (0.705, 1.340)	0.86	0.940 (0.682, 1.297)	0.71
Clinic visit for uncomplicated malaria	Intervention	0.374 (0.309, 0.453)	<0.0001	0.374 (0.309, 0.453)	<0.0001
	Post-intervention	1.008 (0.865, 1.173)	0.92	1.007 (0.866, 1.172)	0.92
	Overall	0.663 (0.584, 0.753)	<0.0001	0.662 (0.584, 0.751)	<0.0001
Clinic visit for uncomplicated malaria with parasitaemia >5000/µL	Intervention	0.439 (0.346, 0.556)	<0.0001	0.441 (0.348, 0.557)	<0.0001
	Post-intervention	0.900 (0.757, 1.070)	0.23	0.891 (0.751, 1.057)	0.19
	Overall	0.720 (0.617, 0.839)	<0.0001	0.719 (0.618, 0.836)	<0.0001
Clinic visit for any illness	Intervention	0.761 (0.680, 0.853)	<0.0001	0.757 (0.677, 0.847)	<0.0001
	Post-intervention	1.066 (0.955, 1.190)	0.25	1.058 (0.949, 1.179)	0.31
	Overall	0.910 (0.833, 0.995)	0.039	0.904 (0.828, 0.986)	0.023
Clinic visit for illness unrelated to malaria	Intervention	1.176 (1.006, 1.374)	0.042	1.169 (1.001, 1.364)	0.049
	Post-intervention	1.128 (0.951, 1.338)	0.17	1.117 (0.942, 1.324)	0.20
	Overall	1.160 (1.019, 1.320)	0.025	1.150 (1.011, 1.307)	0.033

IRR=Incidence rate ratio obtained by negative binomial regression. The source studies contributing to this analysis included Phiri et al, 2012;[6] and Kwambai et al, 2020.[5]

Table S6: Impact of different transformations of continuous covariates on the overall model fit and the adjusted effect size estimate for the effect of PDMC on all-cause readmissions

Age	Model	AIC	Hazard ratio (95% CI)	Protective efficacy (95% CI)	P-value
	Categorical*	288	0.449 (0.360, 0.561)	55.1% (43.9%, 64.0%)	<0.0001
	Linear	288	0.453 (0.363, 0.565)	54.7% (43.5%, 63.7%)	<0.0001
	Quadratic	288	0.454 (0.364, 0.566)	54.6% (43.4%, 63.6%)	<0.0001
	Cubic	286	0.454 (0.364, 0.566)	54.6% (43.4%, 63.6%)	<0.0001
	FP1: Power (0.5)	289	0.452 (0.363, 0.564)	54.8% (43.6%, 63.7%)	<0.0001
	FP1: Power (0)	289	0.452 (0.362, 0.564)	54.8% (43.6%, 63.8%)	<0.0001
	FP1: Power(-0.5)	289	0.453 (0.363, 0.565)	54.7% (43.5%, 63.7%)	<0.0001
	FP1: Power(-1)	288	0.454 (0.364, 0.566)	54.6% (43.4%, 63.6%)	<0.0001
	FP1: Power(-2)	288	0.454 (0.364, 0.566)	54.6% (43.4%, 63.6%)	<0.0001

Dose	Model	AIC	Hazard ratio (95% CI)	Protective efficacy (95% CI)	P-value
	Terciles (categorical)	286	0.447 (0.358, 0.557)	55.3% (44.3%, 64.2%)	<0.0001
	Terciles (ordinal)	288	0.449 (0.360, 0.561)	55.1% (43.9%, 64.0%)	<0.0001
	Linear	289	0.450 (0.361, 0.562)	55.0% (43.8%, 63.9%)	<0.0001
	Quadratic	287	0.449 (0.360, 0.561)	55.1% (43.9%, 64.0%)	<0.0001
	Cubic	287	0.449 (0.360, 0.561)	55.1% (43.9%, 64.0%)	<0.0001
	FP1: Power (0.5)	290	0.451 (0.361, 0.563)	54.9% (43.7%, 63.9%)	<0.0001
	FP1: Power (0)	291	0.451 (0.361, 0.563)	54.9% (43.7%, 63.9%)	<0.0001
	FP1: Power(-0.5)	292	0.451 (0.361, 0.562)	54.9% (43.8%, 63.9%)	<0.0001
	FP1: Power(-1)	293	0.450 (0.361, 0.562)	55.0% (43.8%, 63.9%)	<0.0001
	FP1: Power(-2)	293	0.449 (0.360, 0.560)	55.1% (44.0%, 64.0%)	<0.0001

AIC= Akaike Information Criterion. Dose=dose in mg/kg. FP1=fractional polynomial degree 1 with different sets of powers

*Age categories <12, 12-23, 24-35, 36-47, >=48 months

Table S7: Number, proportion and reasons for censoring by endpoint

Endpoint	Period	>=Event	Died*	LTFU	No event before the end of the study period	total censored	total followed	Studies
Readmission or death from any cause	Overall	536 (22.1%)	NA	446 (23.6%)	1440 (76.4%)	1886	2422	4-6
	Intervention	307 (12.7%)	NA	49 (2.3%)	2066 (97.7%)	2115	2422	5,6
	Post-intervention	315 (13.5%)	NA	434 (21.5%)	1582 (78.5%)	2016	2331	5,6
Readmission for any reason	Overall	489 (20.2%)	47 (2.4%)	446 (23.1%)	1440 (74.5%)	1933	2422	5,6
	Intervention	308 (8.8%)	19 (0.6%)	49 (1.5%)	3129 (97.9%)	3197	3505	4-6
	Post-intervention	287 (12.3%)	28 (1.4%)	403 (19.7%)	1613 (78.9%)	2044	2331	5,6
Readmission for severe anaemia of any cause	Overall	216 (8.9%)	47 (2.1%)	517 (23.4%)	1642 (74.4%)	2206	2422	5,6
	Intervention	138 (3.9%)	19 (0.6%)	51 (1.5%)	3297 (97.9%)	3367	3505	4-6
	Post-intervention	117 (5.0%)	28 (1.3%)	478 (21.6%)	1703 (77.1%)	2209	2326	5,6
Readmission for severe malarial anaemia	Overall	155 (6.4%)	47 (2.1%)	538 (23.7%)	1682 (74.2%)	2267	2422	5,6
	Intervention	95 (2.7%)	19 (0.6%)	52 (1.5%)	3339 (97.9%)	3410	3505	4-6
	Post-intervention	80 (3.5%)	28 (1.3%)	490 (22.0%)	1714 (76.8%)	2232	2312	5,6
Readmission for severe malaria or anaemia	Overall	375 (15.5%)	47 (2.3%)	485 (23.7%)	1515 (74.0%)	2047	2422	5,6
	Intervention	220 (9.1%)	19 (0.9%)	50 (2.3%)	2133 (96.9%)	2202	2422	5,6
	Post-intervention	220 (9.4%)	28 (1.3%)	457 (21.6%)	1626 (77.0%)	2111	2331	5,6
Readmission for severe malaria	Overall	324 (13.4%)	47 (2.2%)	502 (23.9%)	1549 (73.8%)	2098	2422	5,6
	Intervention	191 (7.9%)	19 (0.9%)	51 (2.3%)	2161 (96.9%)	2231	2422	5,6
	Post-intervention	183 (7.9%)	28 (1.3%)	471 (21.9%)	1649 (76.8%)	2148	2331	5,6
Readmission for severe malarial anaemia with parasitaemia >5000/µL	Overall	74 (3.1%)	47 (2.0%)	553 (23.6%)	1748 (74.4%)	2348	2422	5,6
	Intervention	48 (1.4%)	19 (0.5%)	52 (1.5%)	3386 (97.9%)	3457	3505	4-6
	Post-intervention	34 (1.5%)	28 (1.2%)	500 (21.9%)	1750 (76.8%)	2278	2312	5,6
Readmission for other reasons	Overall	149 (6.2%)	47 (2.1%)	522 (23.0%)	1704 (75.0%)	2273	2422	5,6
	Intervention	82 (3.4%)	19 (0.8%)	51 (2.2%)	2270 (97.0%)	2340	2422	5,6
	Post-intervention	76 (3.3%)	28 (1.2%)	483 (21.4%)	1744 (77.3%)	2255	2331	5,6
Clinic visit for uncomplicated malaria	Overall	835 (34.5%)	47 (3.0%)	365 (23.0%)	1175 (74.0%)	1587	2422	5,6
	Intervention	683 (19.5%)	19 (0.7%)	46 (1.6%)	2757 (97.7%)	2822	3505	4-6
	Post-intervention	570 (24.5%)	28 (1.6%)	363 (20.6%)	1370 (77.8%)	1761	2331	5,6
Clinic visit for uncomplicated malaria with parasitaemia >5000/µL	Overall	580 (24.0%)	47 (2.6%)	401 (21.9%)	1387 (75.6%)	1835	2415	5,6
	Intervention	379 (10.8%)	19 (0.6%)	51 (1.6%)	3049 (97.8%)	3119	3498	4-6
	Post-intervention	450 (19.5%)	28 (1.5%)	370 (19.9%)	1464 (78.6%)	1862	2312	5,6
Clinic visit for any illness	Overall	1419 (58.6%)	47 (4.7%)	216 (21.5%)	740 (73.8%)	1003	2422	5,6
	Intervention	1285 (36.7%)	19 (0.9%)	42 (1.9%)	2159 (97.3%)	2220	3505	4-6
	Post-intervention	1004 (43.1%)	28 (2.1%)	246 (18.5%)	1053 (79.4%)	1327	2331	5,6
Clinic visit for illness unrelated to malaria	Overall	851 (35.1%)	47 (3.0%)	362 (23.0%)	1162 (74.0%)	1571	2422	5,6
	Intervention	805 (23.0%)	19 (0.7%)	48 (1.8%)	2633 (97.5%)	2700	3505	4-6
	Post-intervention	507 (21.8%)	28 (1.5%)	380 (20.8%)	1416 (77.6%)	1824	2331	5,6

NA=Not applicable because death was part of the composite endpoint "Readmission or death from any cause".

Supplemental figures

Figure S1: Sensitivity analysis of all-cause readmissions using alternative models

Model type	HR or IRR (95% CI)	P-value
**Extended Cox regression**		
Prentice Williams Peterson - Total Time (primary analysis)	0.45 (0.36, 0.56)	<0.0001
Prentice Williams Peterson - Gap Time	0.45 (0.36, 0.56)	<0.0001
Andersen Gill	0.42 (0.34, 0.53)	<0.0001
**Count models**		
Negative binomial regression	0.42 (0.34, 0.53)	<0.0001
Poisson regression	0.40 (0.32, 0.49)	<0.0001
Zero inflated Poisson regression	0.43 (0.34, 0.54)	<0.0001

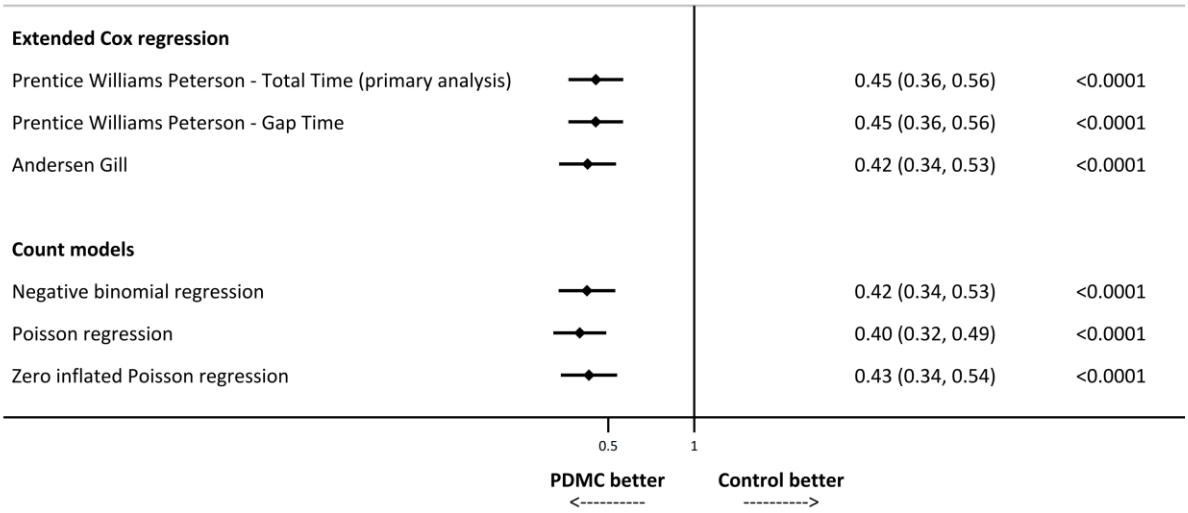

IRR=incidence rate ratio. HR=hazard ratio.

Figure S2: Kaplan Meier curves and treatment effect on time to first and second all-cause readmission using standard Cox regression

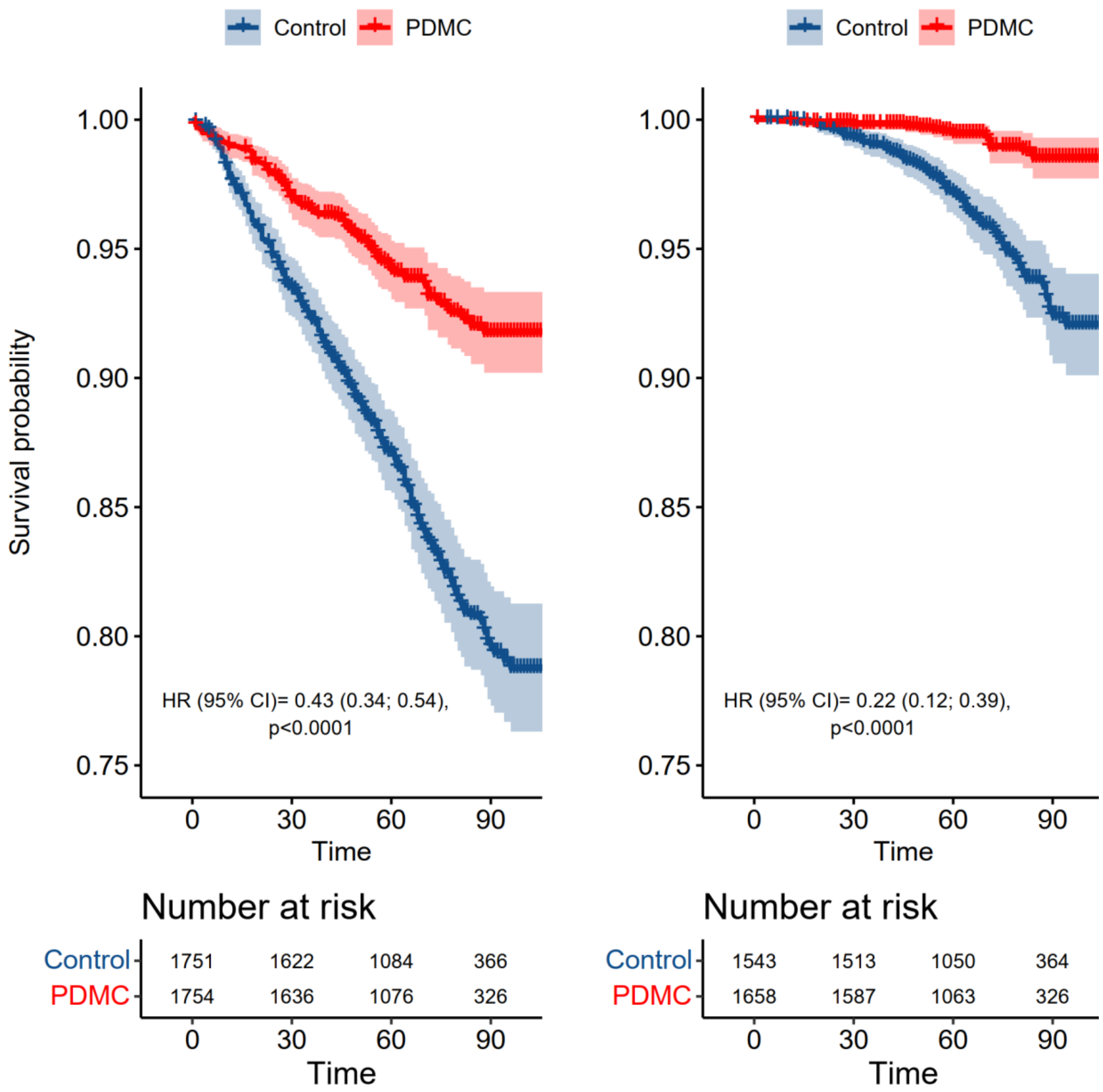

Left panel: time to first readmission. Right panel: time to second readmission. Time in days

Figure S3: Interaction by study for other secondary outcomes (three trials)

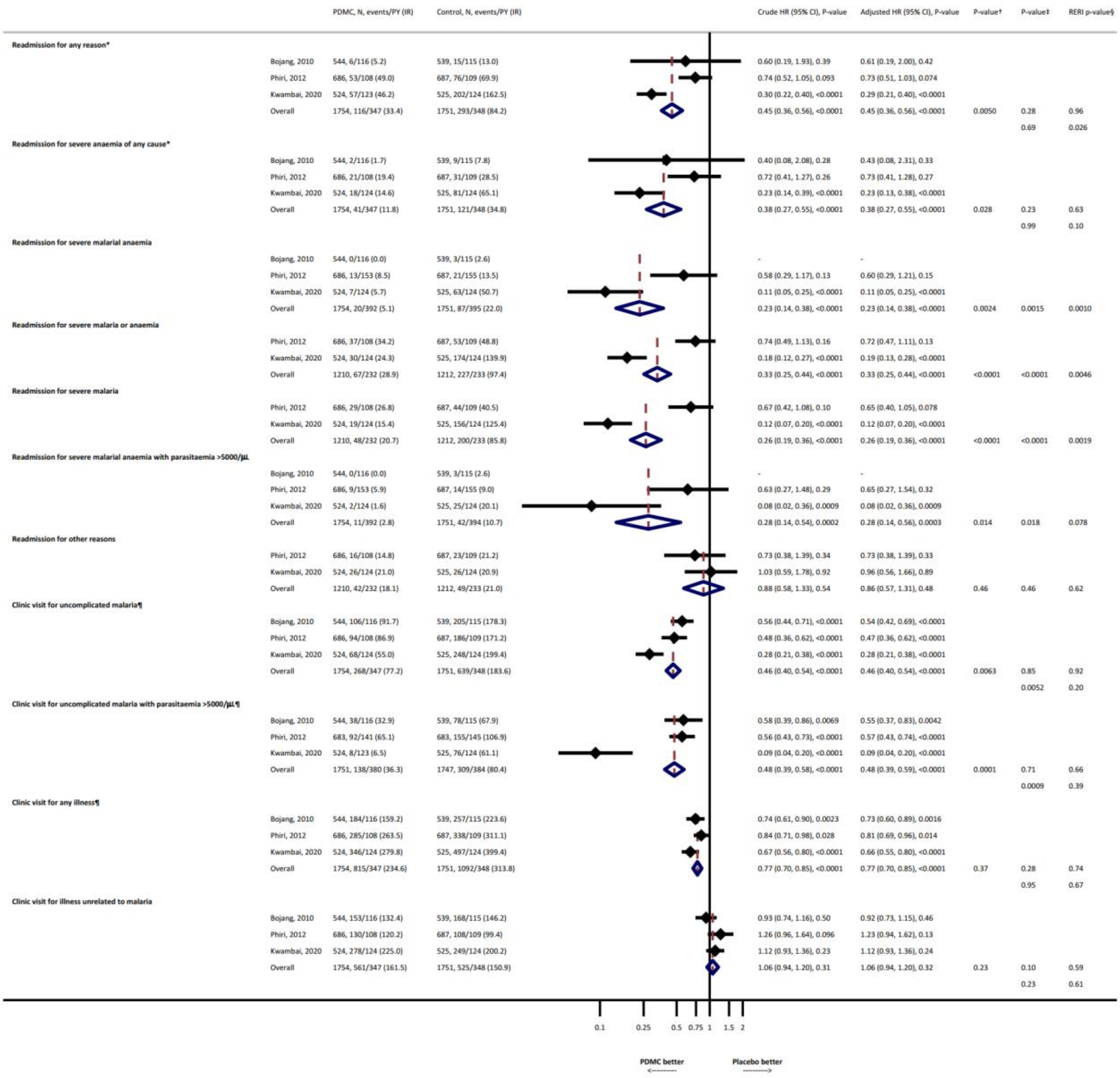

PDMC=Post-discharge malaria chemoprevention. N=number of children contributing, n=number of events. PY=person-years, IR=incidence rate per 100 person-years. HR=hazard ratio. The source studies contributing to this analysis were Bojang et al, 2010[8]; Phiri et al, 2012;[9] and Kwambai et al, 2020.[10]

*The numbers of readmissions for any reason and severe anaemia in the study by Bojang et al.[8] are higher than the number reported in the source publication because, in the current analysis, children with severe anaemia (Hb<5 g/dL) who were treated as outpatients (2 and 9 in the PDMC and placebo group, respectively) were included under the readmission outcomes for consistency with the other two trials. †P-value for differences in treatment effect by study assessed by the ANOVA function on the full and reduced model. ‡P-value for the multiplicative interaction. § P-value for the addtive interaction. ¶Proportional hazards assumption violated (see appendix p 10 for results by negative binomial regression).

Figure S4: Interaction by study period for other secondary outcomes (two trials)

Outcome	Period	PDMC, N, events/PY (IR)	Control, N, events/PY (IR)	Hazard Ratio (95% CI)	P-value	Adjusted HR (95% CI)	P-value	Interaction HR (95% CI)	P-value†	RERI (95% CI)	P-value‡	NNT (95% CI)
**Readmission or death from any cause**												
	Intervention	1210, 111/232 (47.9)	1212, 288/233 (123.6)	0.43 (0.34, 0.53)	<0.0001	0.42 (0.33, 0.52)	<0.0001					6.7 (4.6, 12.4)
	Post-intervention*	1170, 199/280 (71.2)	1161, 182/276 (66.0)	1.10 (0.90, 1.36)	0.35	1.07 (0.87, 1.32)	0.50					
	Overall	1210, 309/513 (60.3)	1212, 471/510 (92.3)					0.71 (0.60, 0.83)	<0.0001	-2.20 (-3.71, -0.68)	0.012	8.8 (5.5, 21.6)
**Readmission for any reason**												
	Intervention	1210, 110/232 (47.5)	1212, 278/233 (119.3)	0.44 (0.35, 0.55)	<0.0001	0.43 (0.34, 0.54)	<0.0001					7.2 (4.8, 14.2)
	Post-intervention*	1170, 185/280 (66.2)	1161, 175/276 (63.4)	1.06 (0.86, 1.32)	0.58	1.03 (0.84, 1.28)	0.76					
	Overall	1210, 295/513 (57.5)	1212, 453/510 (88.8)					0.71 (0.60, 0.83)	<0.0001	-1.84 (-3.18, -0.51)	0.026	9.0 (5.6, 23.3)
**Readmission for severe anaemia of any cause**												
	Intervention	1210, 39/232 (16.8)	1212, 112/233 (48.1)	0.37 (0.26, 0.54)	<0.0001	0.37 (0.26, 0.54)	<0.0001					14.7 (10.1, 27.3)
	Post-intervention*	1168, 63/279 (22.6)	1158, 80/275 (29.1)	0.87 (0.62, 1.23)	0.43	0.83 (0.59, 1.18)	0.30					
	Overall	1210, 102/513 (19.9)	1212, 192/510 (37.6)					0.60 (0.46, 0.77)	0.0015	-1.53 (-3.40, 0.34)	0.12	13.9 (8.9, 31.6)
**Readmission for severe malarial anaemia**												
	Intervention*	1210, 20/276 (7.2)	1212, 84/280 (30.0)	0.24 (0.14, 0.39)	<0.0001	0.24 (0.14, 0.39)	<0.0001					16.3 (11.6, 27.7)
	Post-intervention*	1160, 41/277 (14.8)	1152, 48/273 (17.6)	0.87 (0.57, 1.32)	0.51	0.82 (0.54, 1.25)	0.35					
	Overall	1210, 60/514 (11.7)	1212, 132/510 (25.9)					0.48 (0.35, 0.66)	0.0002	-3.50 (-7.87, 0.87)	0.019	17.6 (11.5, 37.4)
**Readmission for severe malaria or anaemia**												
	Intervention	1210, 67/232 (28.9)	1212, 227/233 (97.4)	0.33 (0.25, 0.44)	<0.0001	0.33 (0.25, 0.44)	<0.0001					7.6 (5.1, 15.2)
	Post-intervention*	1170, 137/280 (49.0)	1161, 134/276 (48.6)	1.04 (0.81, 1.34)	0.75	1.01 (0.78, 1.30)	0.95					
	Overall	1210, 204/513 (39.8)	1212, 361/510 (70.8)					0.63 (0.52, 0.76)	<0.0001	-2.73 (-4.94, -0.53)	0.010	9.2 (5.8, 22.5)
**Readmission for severe malaria**												
	Intervention	1210, 48/232 (20.7)	1212, 200/233 (85.8)	0.26 (0.19, 0.36)	<0.0001	0.26 (0.19, 0.36)	<0.0001					7.9 (5.2, 17.3)
	Post-intervention*	1170, 115/280 (41.1)	1161, 102/276 (37.0)	1.09 (0.82, 1.44)	0.57	1.06 (0.80, 1.41)	0.68					
	Overall	1210, 163/513 (31.8)	1212, 302/510 (59.2)					0.59 (0.48, 0.73)	<0.0001	-4.99 (-9.27, -0.72)	0.0007	10.3 (6.4, 27.1)
**Readmission for severe malarial anaemia with parasitaemia >5000/µL**												
	Intervention	1210, 11/276 (4.0)	1212, 39/279 (14.0)	0.29 (0.15, 0.57)	0.0004	0.29 (0.15, 0.59)	0.0005					35.7 (22.0, 94.9)
	Post-intervention	1160, 16/277 (5.8)	1152, 22/273 (8.0)	0.78 (0.40, 1.51)	0.46	0.76 (0.39, 1.48)	0.42					
	Overall	1210, 26/513 (5.1)	1212, 60/510 (11.8)					0.46 (0.28, 0.75)	<0.0001	-2.61 (-7.65, 2.44)	0.10	35.0 (19.0, 219.2)
**Readmission for other reasons**												
	Intervention	1210, 42/232 (18.1)	1212, 49/233 (21.0)	0.88 (0.58, 1.33)	0.54	0.86 (0.57, 1.31)	0.48					166.4 (-73.6 ∞ 39.1)§
	Post-intervention	1170, 46/280 (16.5)	1161, 41/276 (14.9)	1.08 (0.71, 1.65)	0.73	1.06 (0.69, 1.62)	0.79					
	Overall	1210, 88/513 (17.2)	1212, 90/510 (17.6)					1.00 (0.74, 1.34)	0.47	-0.23 (-0.98, 0.52)	0.71	445.9 (-43.2 ∞ 36.2)§
**Clinic visit for uncomplicated malaria**												
	Intervention*	1210, 162/232 (69.9)	1212, 434/233 (186.2)	0.37 (0.30, 0.45)	<0.0001	0.37 (0.30, 0.45)	<0.0001					3.4 (2.6, 5.0)
	Post-intervention	1170, 358/280 (128.0)	1161, 353/276 (127.9)	0.99 (0.85, 1.14)	0.85	0.99 (0.85, 1.14)	0.85					
	Overall	1210, 520/513 (101.4)	1212, 787/510 (154.2)					0.66 (0.58, 0.75)	<0.0001	-1.56 (-2.41, -0.71)	0.018	3.9 (2.8, 6.8)
**Clinic visit for uncomplicated malaria with parasitaemia >5000/µL**												
	Intervention*	1207, 100/265 (37.8)	1208, 231/269 (85.8)	0.43 (0.33, 0.54)	<0.0001	0.43 (0.33, 0.54)	<0.0001					5.0 (2.5, 375.2)
	Post-intervention	1160, 264/277 (95.4)	1152, 294/273 (107.6)	0.87 (0.73, 1.03)	0.099	0.86 (0.73, 1.02)	0.077					
	Overall	1207, 338/512 (66.1)	1208, 480/508 (94.4)					0.74 (0.64, 0.85)	<0.0001	-3.24 (-5.39, -1.09)	0.0020	4.4 (-17.4 ∞ 2.0)§
**Clinic visit for any illness**												
	Intervention*	1210, 631/232 (272.2)	1212, 835/233 (358.2)	0.75 (0.66, 0.84)	<0.0001	0.73 (0.65, 0.83)	<0.0001					4.7 (3.2, 9.2)
	Post-intervention	1170, 776/280 (277.4)	1161, 724/276 (262.4)	1.04 (0.94, 1.15)	0.44	1.03 (0.93, 1.14)	0.52					
	Overall	1210, 1407/513 (274.3)	1212, 1560/510 (305.7)					0.90 (0.83, 0.98)	<0.0001	-0.48 (-0.75, -0.22)	0.19	7.1 (3.6, 269.9)
**Clinic visit for illness unrelated to malaria**												
	Intervention	1210, 408/232 (176.1)	1212, 357/233 (153.2)	1.14 (0.98, 1.32)	0.083	1.14 (0.98, 1.32)	0.091					-15.8 (-7.7 ∞ 358.4)§
	Post-intervention	1170, 353/280 (126.2)	1161, 309/276 (112.0)	1.11 (0.94, 1.31)	0.21	1.10 (0.94, 1.30)	0.25					
	Overall	1210, 769/513 (150.0)	1212, 668/510 (130.9)					1.14 (1.01, 1.29)	0.98	0.04 (-0.18, 0.26)	0.90	-10.0 (-323.0, -5.1)

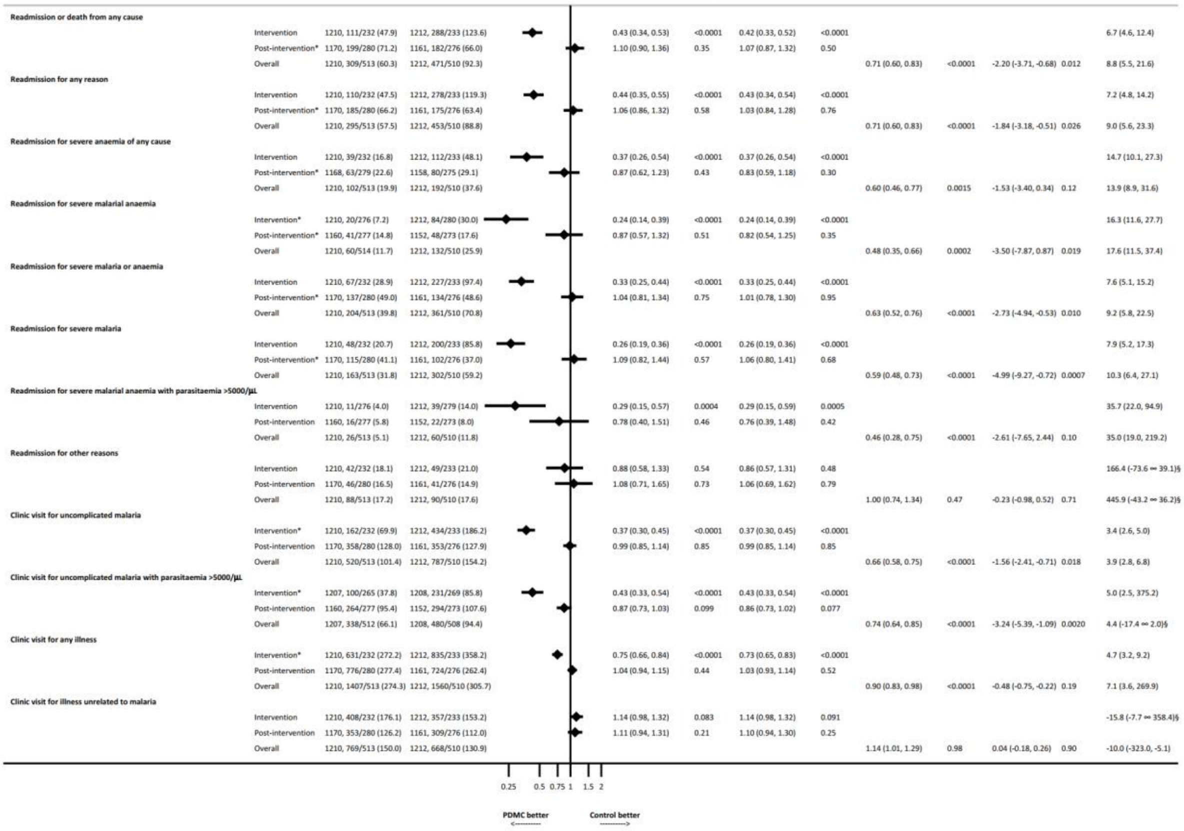

PDMC=Post-discharge malaria chemoprevention. N=number of children contributing, n=number of events. PY=person-years, IR=incidence rate per 100 person-years. HR=hazard ratio. CI=confidence interval. Interaction HR=ratio of hazard ratios (muliplicative interaction). RERI= relative excess risk due to interaction (additive interaction). NNT number needed to treat to avert one event during the intervention period or overall (intervention and post-intervention periods pooled). The two source studies contributing to this analysis were Phiri et al, 2012[9] and Kwambai et al, 2020.[10] *Proportional hazards assumption violated (see appendix p 9 for results by negative binomial regression). †P-value for the multiplicative interaction. ‡ P-value for the additive interaction. § Left CI illustrates NNT to harm (NNH) and the right CI illustrates NNT to benefit (NNT). The ∞ symbol illustrates that the NNH or NNT include infinity.

Supplemental references

1. World Health Organisation; Global Malaria Programme. World Malaria Report 2020: 20 years of global progress & challenges; 2020.
2. Minozzi S, Cinquini M, Gianola S, Gonzalez-Lorenzo M, Banzi R. The revised Cochrane risk of bias tool for randomized trials (RoB 2) showed low interrater reliability and challenges in its application. *J Clin Epidemiol* 2020; **126**: 37-44.
3. Higgins J, Savović J, Page MJ, Elbers RG, Sterne JAC. Chapter 8: Assessing risk of bias in randomized trials. 2021. https://training.cochrane.org/handbook/current.
4. Bojang KA, Milligan PJ, Conway DJ, et al. Prevention of the recurrence of anaemia in Gambian children following discharge from hospital. *PloS one* 2010; **5**(6): e11227.
5. Kwambai TK, Dhabangi A, Idro R, et al. Malaria Chemoprevention in the Postdischarge Management of Severe Anemia. *N Engl J Med* 2020; **383**(23): 2242-54.
6. Phiri K, Esan M, van Hensbroek MB, Khairallah C, Faragher B, ter Kuile FO. Intermittent preventive therapy for malaria with monthly artemether-lumefantrine for the post-discharge management of severe anaemia in children aged 4-59 months in southern Malawi: a multicentre, randomised, placebo-controlled trial. *The Lancet Infectious diseases* 2012; **12**(3): 191-200.
7. Fox GA, Negrete-Yankelevich S, Sosa VJ. Ecological Statistics: Contemporary theory and application. Oxford and New York: Oxford University Press; 2015.
8. Lin E, Tong T, Chen Y, Wang Y. Fixed-effects model: the most convincing model for meta-analysis with few studies. *arXiv API* 2020.
9. Borenstein M, Hedges LS, Higgens JPT, Rothstein HR. Chapter 13: Fixed-Effect Versus Random-Effects Models. Introduction to meta-analysis. Chichester, United Kingdom: John Wiley & Sons, Ltd; 2009.
10. Cornell JE, Mulrow CD, Localio R, et al. Random-effects meta-analysis of inconsistent effects: a time for change. *Ann Intern Med* 2014; **160**(4): 267-70.
11. Rogers JK, Jhund PS, Perez AC, et al. Effect of rosuvastatin on repeat heart failure hospitalizations: the CORONA Trial (Controlled Rosuvastatin Multinational Trial in Heart Failure). *JACC Heart Fail* 2014; **2**(3): 289-97.
12. Prentice RL, Williams BJ, Peterson AV. On the regression analysis of multivariate failure time data. *Biometrika* 1981; **68**(2): 373-9.
13. Ozga AK, Kieser M, Rauch G. A systematic comparison of recurrent event models for application to composite endpoints. *BMC Med Res Methodol* 2018; **18**(1): 2.
14. Fávero LP, Belfiore P. OVERDISP: Stata module to detect overdispersion in count-data models using Stata. 2018.
15. Knol MJ, VanderWeele TJ. Recommendations for presenting analyses of effect modification and interaction. *International journal of epidemiology* 2012; **41**(2): 514-20.
16. Richardson DB, Kaufman JS. Estimation of the Relative Excess Risk Due to Interaction and Associated Confidence Bounds. *American Journal of Epidemiology* 2009; **169**(6): 756-60.
17. Schünemann HJ, Oxman AD, Higgins JPT, et al. Chapter 11: Presenting results and 'Summary of findings' tables. In: Higgins JPT, Green S, eds. Cochrane Handbook for Systematic Reviews of Interventions Version 501 [updated September 2008] The Cochrane Collaboration: John Wiley & Sons, Ltd; 2008.
18. Maitland K, Olupot-Olupot P, Kiguli S, et al. Co-trimoxazole or multivitamin multimineral supplement for post-discharge outcomes after severe anaemia in African children: a randomised controlled trial. *The Lancet Global health* 2019; **7**(10): e1435-e47.
19. Altman DG. Confidence intervals for the number needed to treat. *Bmj* 1998; **317**(7168): 1309-12.
20. Deeks JJ, Higgins JP, Altman DG. Analysing data and undertaking meta-analyses. In: Higgins JP, Green S, eds. Cochrane handbook for systematic reviews of interventions (version 510): The Cochrane Collaboration; 2011.

21. Flegg JA, Humphreys GS, Montanez B, et al. Spatiotemporal spread of Plasmodium falciparum mutations for resistance to sulfadoxine-pyrimethamine across Africa, 1990 – 2020. *Plos Comp Biol* 2022 (in press).
